# Dissection of the gut microbiota in mothers and children with chronic *Trichuris trichiura* infection in Pemba Island, Tanzania

**DOI:** 10.1186/s13071-021-04580-1

**Published:** 2021-01-19

**Authors:** Hongliang Chen, Matteo Mozzicafreddo, Elisa Pierella, Vanessa Carletti, Angela Piersanti, Said M. Ali, Shaali M. Ame, Chunfeng Wang, Cristina Miceli

**Affiliations:** 1 College of Veterinary Medicine, Jilin Provincial Engineering Research Center of Animal Probiotics, Key Laboratory of Animal Production and Product Quality Safety of Ministry of Education, Jilin Agricultural University, Changchun, China; 2 School of Biosciences and Veterinary Medicine, University of Camerino, 62032 Camerino, Italy; 3 Public Health Laboratory Ivo de Carneri, Pemba Island, Chake Chake, Tanzania

**Keywords:** Neglected tropical disease, Soil-transmitted helminthiases, Gut microbiota, 16S rRNA

## Abstract

**Background:**

Soil-transmitted helminthiases are important neglected tropical diseases that result in a notably high number of disability-adjusted life years worldwide. Characterizing the interactions between the human intestinal microbiome and helminths is of interest in the development of alternative treatments that do not rely on chemotherapeutics and do not lead to drug resistance.

**Methods:**

We recruited and obtained fecal samples from 32 pairs of mothers and children on Pemba Island and monitored their intestinal microbiota using 16S rRNA gene sequencing.

**Results:**

We observed that microbial changes occur in the gut microbiota of infected mothers and children. Some short-chain fatty acid (SCFA)-producing bacteria and carbohydrate-degrading bacteria exhibited lower abundance in the infected individuals. Potentially pathogenic *Campylobacter* and proinflammatory *Methanobrevibacter* in infected mothers and opportunistic *Enterococcus* in infected children exhibited greater abundance.

**Conclusions:**

Our findings could reveal the microbiota profiling in *T. trichiura*-infected individuals, indicate the potential roles of key microbiota in the host and aid to the development of novel strategies to control *T. trichiura* infection.

**Graphic abstract:**

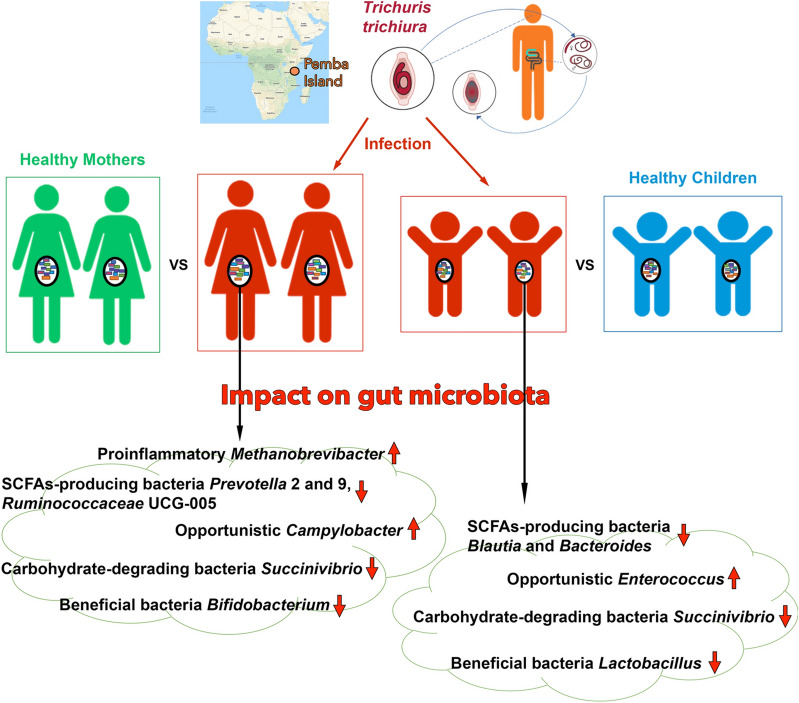

## Background

Soil-transmitted helminthiases (STHs) are among the most widespread neglected tropical diseases (NTDs) in low-income populations in developing regions of Africa, Asia and the Americas. Four major nematode groups, including roundworms (*Ascaris lumbricoides*), whipworms (*Trichuris trichiura*), hookworms (*Ancylostoma duodenale* and *Necator americanus*), and threadworms (*Strongyloides stercoralis*), are responsible for STHs. More than 1.5 billion people are infected by soil-transmitted helminths, and 4.98 million disability-adjusted life years (DALYs) are caused by soil-transmitted helminths worldwide [1, 2]. Chronic STHs can lead to anemia, malnutrition, asthenia, abdominal pain, diarrhea, stunted growth, and cognitive and developmental impairment [3]. These nematodes penetrate the intestinal mucosa, jeopardize the intestinal epithelium and disrupt gut homeostasis [4]. STHs have been reported to increase the abundance of potential intestinal pathobionts and disrupt the structure of the gut microbial community [5]. Currently, chemotherapeutic drugs are the global strategy to control STHs, but this approach has led to an unavoidable increase in drug resistance [6]. With the emergence of anthelmintic resistance, alternative treatments are urgently required to reach the goal of the WHO to eliminate STHs [7].

The gut microbiota has been demonstrated to play pivotal roles in host health, serving such purposes as absorbing nutrients from food, modulating metabolism, conferring resistance to colonization by some enteric pathogens and promoting the development of the immune system [8, 9]. A growing body of research has focused on helminth-microbiota interactions: for instance, some probiotic strains of *Bifidobacterium* and *Lactobacillus* have been evaluated to modulate helminth infection in vivo and in vitro [10–13]. Moreover, the impact of low-intensity, chronic helminth infection on microbial communities has been commonly reported to increase microbial alpha diversity [14–17]. In contrast, high-intensity, acute infections have been often associated to gut dysbiosis, characterized by reduced alpha diversity and an increase in pro-inflammatory and opportunistic pathogens [18]. New perspectives of helminth therapies are proposed because helminth infection may contribute positively to gut homeostasis by promoting microbial richness and evenness in individuals with chronic inflammatory disorders [17, 19, 20]; therefore, it would be of interest to investigate whether helminth-induced immune modulation is related to alterations in the microbiota. Analyses of gut microbiota-mediated modulations in the worm removal and egg production of nematodes are also of interest to suggest the use of probiotics as a promising alternative means of controlling STHs by improving human health [21].

Some investigations of the gut microbiota of humans naturally infected by STHs have been reported. Jenkins et al. [15] reported that *S. stercoralis* infection was associated with decreased abundance of *Bacteroides eggerthii*, *Clostridium celatum* and *Bifidobacterium bifidum* in a cohort of elderly Italian volunteers by 16S rRNA gene sequencing. Lee et al. [14] also found that *Bifidobacterium* exhibited lower abundance and the Paraprevotellaceae exhibited expanded abundance in *T. trichiura*-infected individuals in Malaysia. In this study, we compared the gut microbial community of *T. trichiura*-colonized and non-colonized individuals from Pemba Island, using 16 rRNA gene sequencing. Pemba Island is highly endemic for STHs; thus, this location provides a good opportunity for characterizing the intestinal microbial community of individuals suffering chronic helminth infection. In particular, we investigated mothers and their children from two rural villages and one town on Pemba Island and identified specific associations between helminth colonization and bacterial species in mothers and children.

## Methods

### Ethics statement

The project was approved by the Zanzibar Medical Ethical Research Committee (protocol number: ZAMREC/001/SEPT/018). Written informed consent was obtained from all subjects enrolled in the study.

### Recruitment of study participants

Women aged 23–45 years and their children aged 1.5–2.6 years from Vitongoji village, Gombani village and Chake-Chake town were recruited for this study on Pemba Island, Tanzania. Thirty-two mother-and-child pairs of volunteers were enrolled. During the visits of the mothers to the sanitary center for their children’s routine examinations, the mothers were interviewed. A questionnaire was prepared for each volunteer. To facilitate reciprocal understanding, interviews were performed in Swahili with the help of nurses and personnel of the Public Health Laboratory Ivo de Carneri (PHL-IdC). The inclusion criteria for all the individuals were as follows: similar nutritional habits (very similar diet, mainly consisting of banana fruit, cassava, rice, cassava leaves as vegetable and dagaa fish, and origin of food), no pregnancy, no HIV, no diarrhea, no fever, no diabetes, no malaria, and no antibiotics or anthelmintic treatment in the previous 3 months. The questionnaire format is included in the Additional file 1: Material S1.

### Fecal sample collection and parasitological analysis

All the participants were provided with sterile containers to collect fecal samples. Each sample was well homogenized and divided into three aliquots. One aliquot was used for parasitological examination directly, one for the DNA extraction, performed upon sample reception, and the third was maintained frozen as a backup. The Mini–FLOTAC technique was utilized for microscopic examination [22]. Two grams of stool sample and 2 ml of 5% formalin were mixed into the conical collector first. After the samples were homogenized sufficiently, flotation solution (saturated sodium chloride) was added to a volume of 40 ml. After another round of homogenization, the samples were added to the two flotation chambers. Finally, after waiting for 10 min, the numbers of eggs per gram of feces were determined under a microscope. Analytic sensitivity could reach ten eggs per gram of feces [23]. This analysis was repeated twice for each sample. All procedures were conducted in the laboratory of the PHL-IdC. After the parasitological analysis was performed, the results were delivered to the enrolled women participants to ensure that they could go to the sanitary center to receive anti-helminth treatment.

### DNA extraction, PCR amplification, 16S rRNA gene sequencing

Total DNA was extracted from fecal samples utilizing the PureLink*™* Microbiome DNA Purification Kit (Invitrogen, Waltham, MA, USA) according to the manufacturer’s instructions. The integrity and concentrations of the obtained DNA were determined by agarose gel electrophoresis and NanoDrop electrophoresis, respectively. Then, the V3-V4 hypervariable region of the 16S rRNA gene was amplified by PCR. The universal primers (forward, Pro341: 5′-TCGTCGGCAGCGTCAGATGTGTATAAGAGACAGCCTACGGGNBGCASCAG-3′; reverse, Pro805: 5′-GTCTCGTGGGCTCGGAGATGTGTATAAGAGACAGGACTACNVGGGTATCTAATCC-3′) were used [24]. PCR was performed in triplicate in 25 μl volumes containing 2.5 μl of 10 × Pyrobest Buffer, 2 μl of 2.5 mM dNTPs, 1 μl of each primer (10 μM), 0.4 U of Pyrobest DNA Polymerase (TaKaRa) and 15 ng of template DNA. The PCR program involved an initial denaturation step at 94 ℃ for 3 min followed by 25 cycles of denaturation at 94 ℃ for 30 s, annealing at 50 ℃ for 30 s and extension at 72 ℃ for 60 s with a final extension phase at 72 ℃ for 7 min.

PCR products were run in an electrophoresis chamber on a 1% agarose gel, and 50 ng of purified DNA extraction of each sample was subsequently prepared and sent to the BMR Genomics company (Padova, Italy) for sequencing. Sequencing libraries were generated using the NEBNext® Ultra™ DNA Library Prep Kit (New England Biolabs, Ipswich, MA, USA) following the manufacturer’s recommendations. Library quality was assessed and sequenced on an Illumina MiSeq platform PE300 platform (Illumina, San Diego, CA, USA).

### Bioinformatics and sequencing data analysis

The original DNA fragments were trimmed of the adapters using Cutadapt (version 1.18; https://cutadapt.readthedocs.io/en/stable/index.html) [25]. FastQC was applied to check the quality of the raw reads after the trimming process (version 0.11.8; http://www.bioinformatics.babraham.ac.uk/projects/fastqc/). The raw reads were processed utilizing QIIME2 (Quantitative Insights Into Microbial Ecology 2, version 2018.11; https://qiime2.org) [26]. Joined sequences were successfully quality-filtered and dereplicated with identification of chimeras. Paired-end reads were merged in QIIME2 using the DADA2 pipeline (version 1.10; https://benjjneb.github.io/dada2/index.html). Sequences were clustered into operational taxonomic units (OTUs) according to the taxonomy assignment through annotation against the SILVA database (version SSU138; https://www.arb-silva.de). Sequences that did not match references in the SILVA database were clustered *de novo* based on pairwise sequence identity (cut-off: 99% sequence similarity). The first selected cluster seed was considered the representative sequence of each OTU. The OTU table with the assigned taxonomy was exported from QIIME2 alongside an unweighted UniFrac distance matrix. Prior to downstream analyses, we removed singleton OTUs. Cumulative-sum scaling (CSS) was applied followed by log2 transformation to account for the non-normal distribution of taxonomic count data. Three metrics of alpha diversity were evaluated using QIIME2; in particular observed OTUs, estimating the microbial richness; Shannon index, estimating species biodiversity; and Faith’s phylogenetic diversity index, measuring community richness incorporating phylogenetic relationships between the features. Differences in alpha diversity were tested by the Wilcoxon rank-sum test. Principal component analysis (PCA) of the OTUs in different groups was performed based on the unweighted UniFrac distance values, and statistical differences in microbial community composition were calculated by the Permutation Multivariate Analysis of Variance (PERMANOVA) function with 999 permutations. Furthermore, a linear discriminant analysis (LDA) effect size (LEfSe) algorithm (http://huttenhower.sph.harvard.edu/galaxy/) was applied to identify the significant microbial differences among all of the groups [27]. This analysis, based on the nonparametric factorial Kruskal-Wallis and on the (unpaired) Wilcoxon rank-sum test, was also validated with a further Student’s t-test using the STAMP software (version 2.1.3). Microbial community metagenome functions were predicted by PICRUSt2 implemented in Qiime2 (Phylogenetic Investigation of Communities by Reconstruction of Unobserved States, version 2019.7; https://github.com/picrust/picrust2/) [28]. PICRUSt2 comparisons between each group were performed using STAMP with Student’s *t*-test (version 2.1.3). *p*-values < 0.05 were considered to be significant. The raw reads were deposited into the NCBI Sequence Read Archive database (SRA accession number: SRP259507, BioProject accession number: PRJNA629760).

## Results

### Characteristics of the participants

Thirty-two pairs of mothers and children were interviewed and provided their consent and the fecal samples. Twenty (12 mothers and 8 children) samples were positive for helminth infection, and 44 (20 mothers and 24 children) samples were negative for helminth infection. Five negative samples were discarded for problems in the DNA extraction or at the sequence level, and three single-*A. lumbricoides*-infected samples were also discarded (sample code M66: 2000 eggs/gram of feces; C46: 4000 eggs/gram of feces; C34: 10 eggs/gram of feces). In the end, 56 samples gave sufficient sequences and were successfully analyzed. Seventeen individuals (11/29 mothers and 6/27 children) carried *T. trichiura* infection, and in only three of these individuals the infection was combined with *A. lumbricoides*. The other 39 individuals (18/29 mothers and 21/27 children) were all negative for helminth infection. Among the 56 samples, there were 12 pairs of uninfected mother-child and 4 pairs of infected mother-child, whereas mothers and children conprising the remaining 16 pairs studied displayed different statuses of infection by *T. trichiura*. The infection status and demographics of the mothers and children are shown in Table 1. Sample codes of all the 32 pairs of mothers and children and infection burdens of helminth-infected participants are shown in Additional file 2: Table S1.

**Table 1:** Characteristics of the participants

	MP	MN	CP	CN
Subject numbers	11	18	6	21
Ages (years)	29.27 ± 4.50	31.39 ± 6.63	1.63 ± 0.50	1.81 ± 0.33
Habited village				
Vitongoji	6	4	3	6
Gombani	3	6	2	6
Chake Chake	2	8	1	9
Helminth infection status	11	0	6	0
Single *T. trichiura*	9	0	5	0
*T. trichiura* + *A. lumbricoides*	2	0	1	0

MP, mother helminth-positive group; MN, mother helminth-negative group; CP, children helminth-positive group; CN, children helminth-negative group

### Dissection of gut microbial profiling in helminth-positive and helminth-negative individuals

The 16S rRNA gene sequencing produced a total of 1,655,316 sequences after assembly and quality filtering from 56 samples, and the average length of the sequences was 410.97 bp. Good's coverage index was > 99%. In total, 6252 OTUs were observed in all four groups, the mother helminth-positive group (MP), mother helminth-negative group (MN), child helminth-positive group (CP) and child helminth-negative group (CN). Rarefaction curves of the OTUs of all samples indicated that there were sufficient data sampling and adequate sequencing depth, and the database of 16S rRNA gene sequences almost completely covered all microbial communities (Additional file 3: Figure S1).

**Fig. 1:**
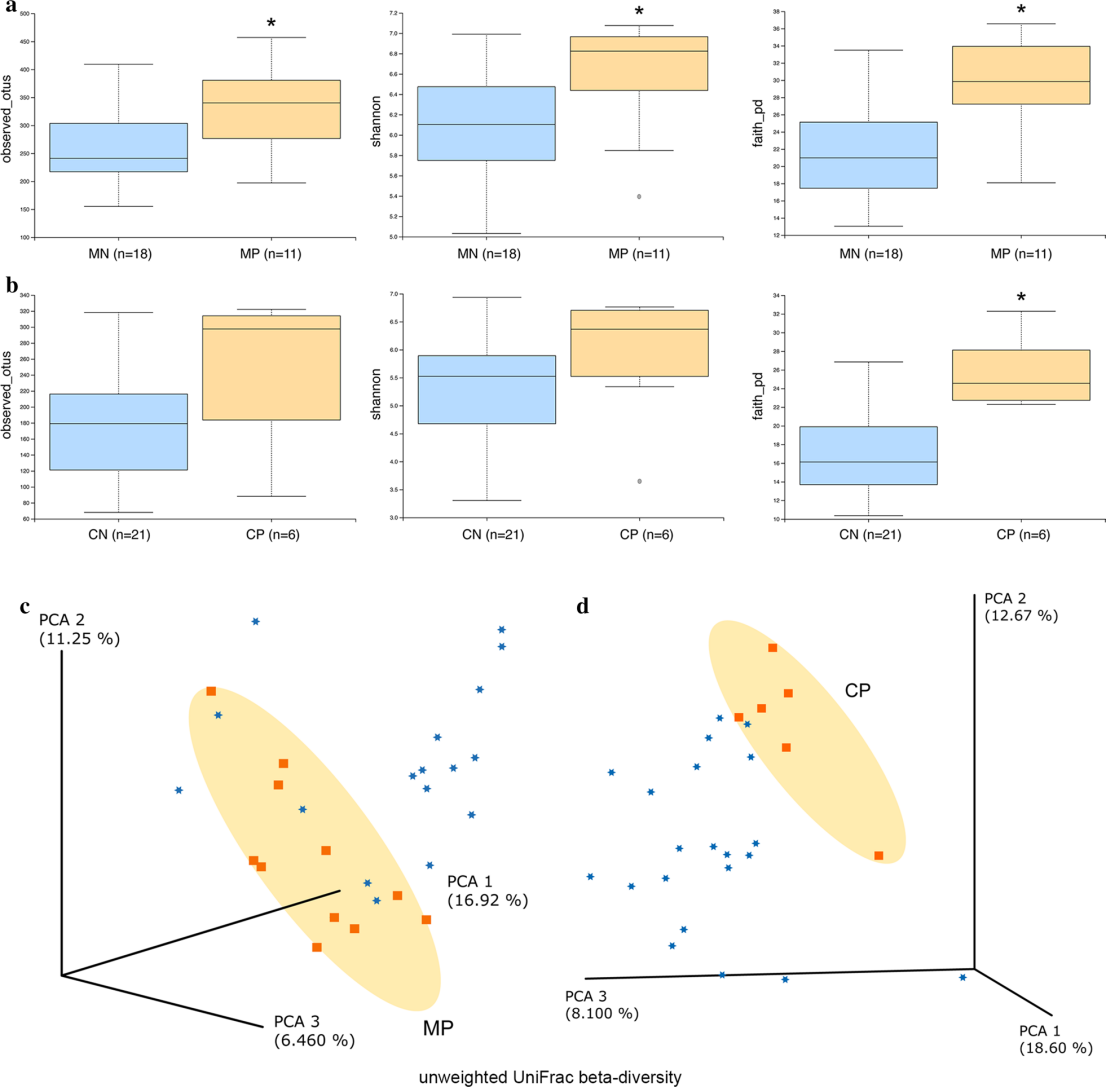
Differences in microbial alpha diversity between helminth-infected and uninfected mothers (**a**) and children (**b**) and principal component analysis (unweighted UniFrac distance) of gut microbial profiles of mothers (**c**) and children (**d**) clustered by infection status (orange squares and blue dots represent samples from infected and uninfected individuals, respectively). *Abbreviations*: MP, mother helminth-positive group; MN, mother helminth-negative group; CP, children helminth-positive group; CN, children helminth-negative group

There was a significant difference in the fecal microbiota alpha diversity between the MN and MP groups. The observed OTUs, Shannon index and Faith’s phylogenetic diversity index in the MP group were significantly higher than those in the MN group, respectively (Kruskal-Wallis H-test: *H* = 3.82, df = 1, *P* = 0.05; *H* = 4.65, df = 1, *P* = 0.03 and *H* = 8.53, df = 1, *P* = 0.003). While the observed OTUs and Shannon index, the latter accounting for both microbial richness and evenness, in group CN were not significantly different from those in the CP group (Kruskal-Wallis H-test: H = 3.06, df = 1, *P* = 0.08 and *H* = 1.36, df = 1, *P* = 0.24), Faith’s phylogenetic diversity index was significantly higher in the CP group than in the CN group (Kruskal-Wallis H-test: *H* = 10.67, df = 1, *P* = 0.001) (Fig. 1a, b).

PCA based on unweighted UniFrac distances showed infection-related clustering of samples collected from mothers and children, respectively (Fig. 1c, d). Moreover, significant infection-associated alterations in gut microbial community structure were observed in mothers (PERMANOVA: pseudo-*F* = 2.05, df = 1, *P* = 0.003) and children (PERMANOVA: pseudo-*F* = 1.96, df = 1, *P* = 0.013), which indicated differences in overall heterogeneity of microbial composition between helminth-positive and helminth-negative groups.

### Microbial taxa analysis

**Fig. 2:**
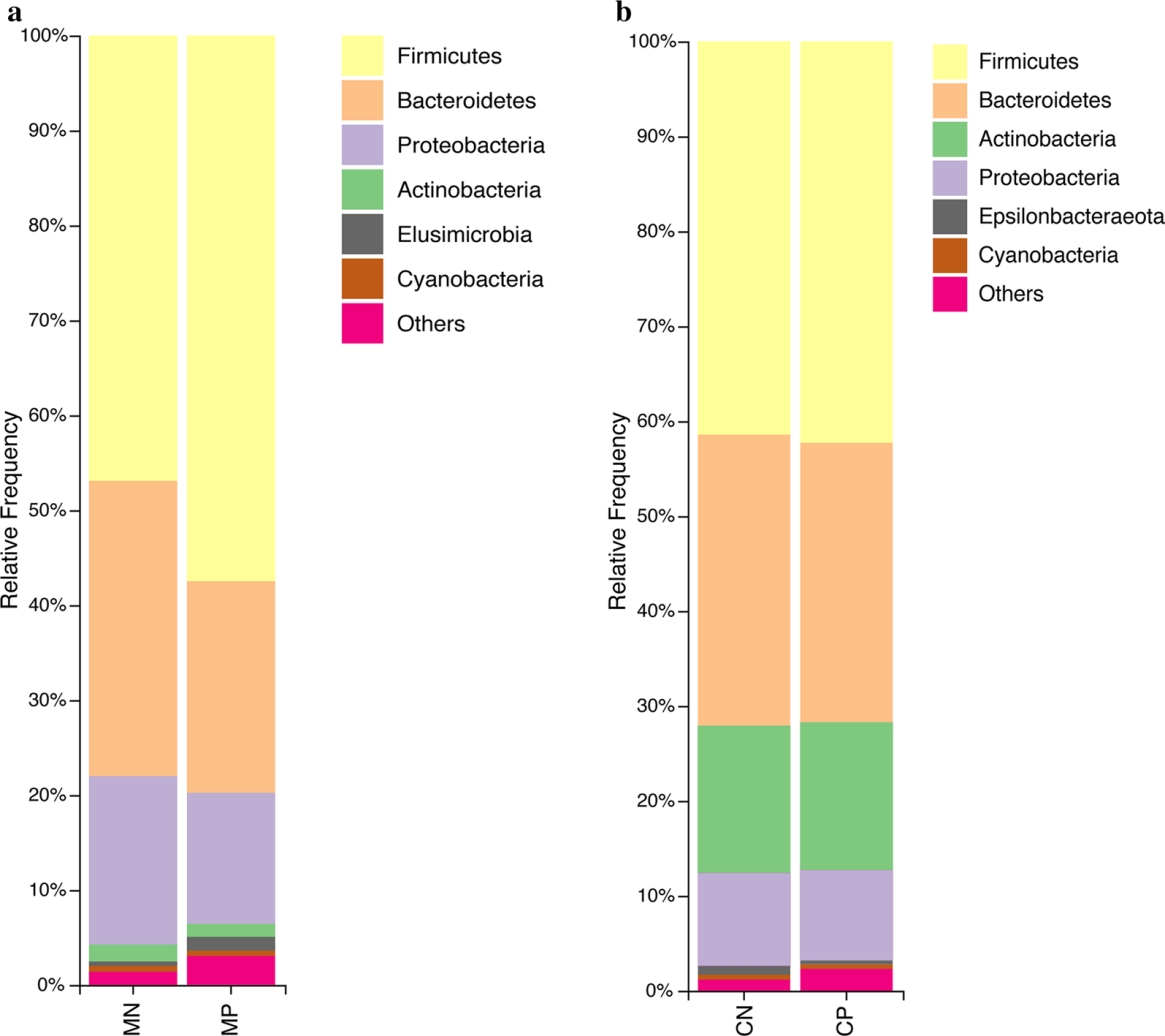
Relative abundances of gut microbial phyla identified in the feces of mothers (**a**) and children (**b**). *Abbreviations*: MP, mother helminth-positive group; MN, mother helminth-negative group; CP, children helminth-positive group; CN, children helminth-negative group; others represent all phyla with relative abundance < 0.5%

Bacterial abundances at the phylum and genus levels were analyzed and compared between infected and non-infected mothers and children. We found that Firmicutes, Bacteroidetes, Proteobacteria and Actinobacteria dominated the intestinal community in group MN at the phylum level with relative abundances of 46.90 (mean abundance)  ± 3.58% (standard error), 31.12 ± 3.76%, 17.73 ± 1.89% and 1.79 ± 0.20%, respectively. In group MP, the relative abundance of Firmicutes was determined to be 57.46 ± 5.24%, while Bacteroidetes, Proteobacteria and Actinobacteria showed lower abundances of 22.31 ± 3.91%, 13.78 ± 1.41% and 1.38 ± 0.15%, respectively (Fig. 2a). The dominant phyla in group CN and their abundances were as follows: Firmicutes (41.42 ± 3.87%), Bacteroidetes (30.65 ±  3.28%), Actinobacteria (15.55 ± 3.51%) and Proteobacteria (9.72 ± 2.80%). Group CP abundances were as shown in Fig. 2b: Firmicutes (42.28 ± 8.22%), Bacteroidetes (29.44 ± 9.27%), Actinobacteria (15.61 ± 9.85%) and Proteobacteria (9.44 ± 3.07%) (Fig. 2b). These results showed that the relative abundance of dominant gut bacterial populations differed between mothers and children at the phylum level. Moreover, in mothers, helminth colonization was associated with a significant enrichment of Firmicutes and significant reductions of Bacteroidetes, Actinobacteria and Proteobacteria in gut microbial populations (Additional file 4: Table S2). In children, variations in the gut microbial composition were not significant at the phylum level.

**Fig. 3:**
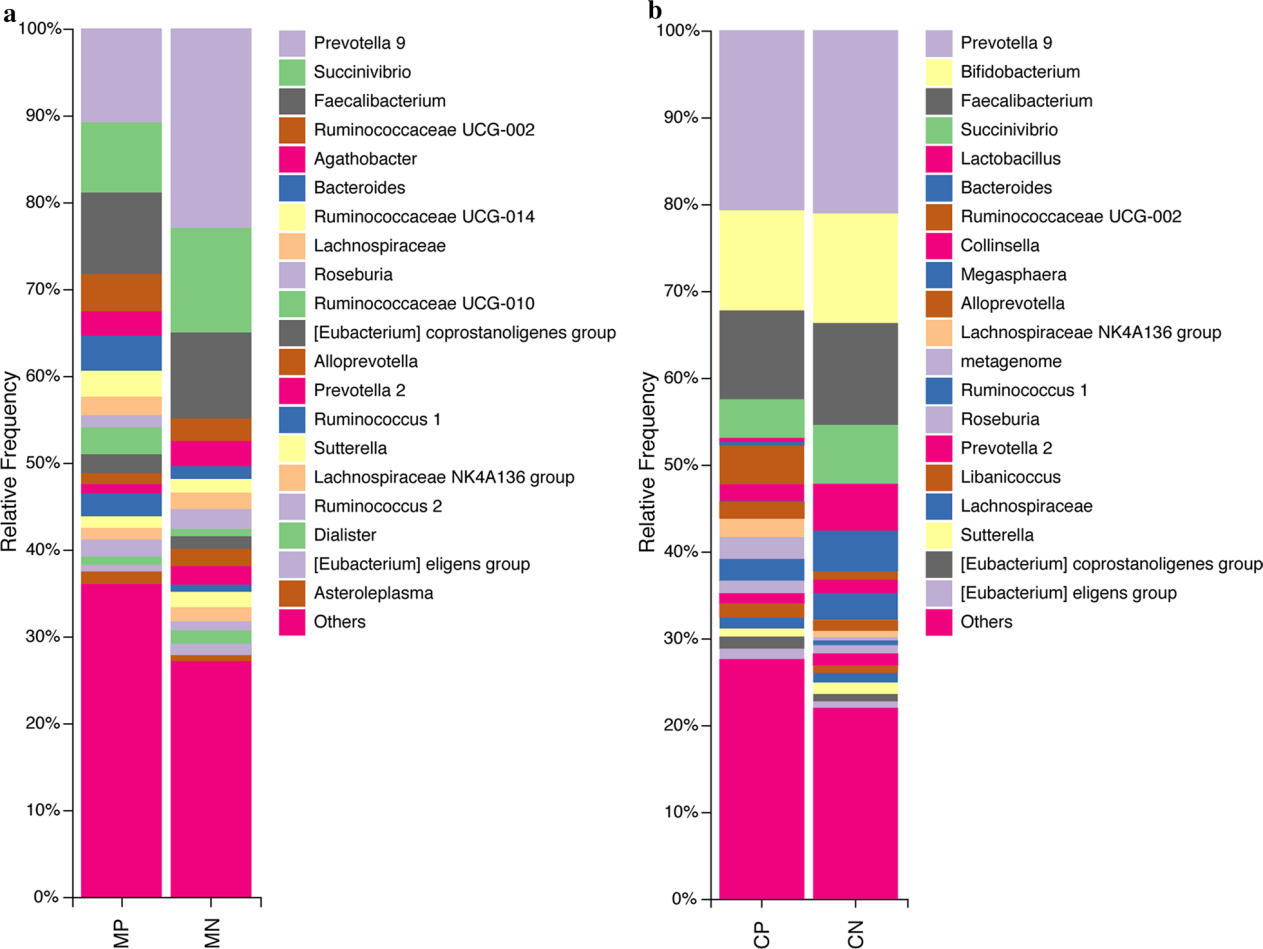
Relative abundances of gut microbial genera identified in the feces of mothers (**a**) and children (**b**). *Abbreviations*: MP, mother helminth-positive group; MN, mother helminth-negative group; CP, children helminth-positive group; CN, children helminth-negative group. Others represent all genera with relative abundance < 0.5%

**Fig. 4:**
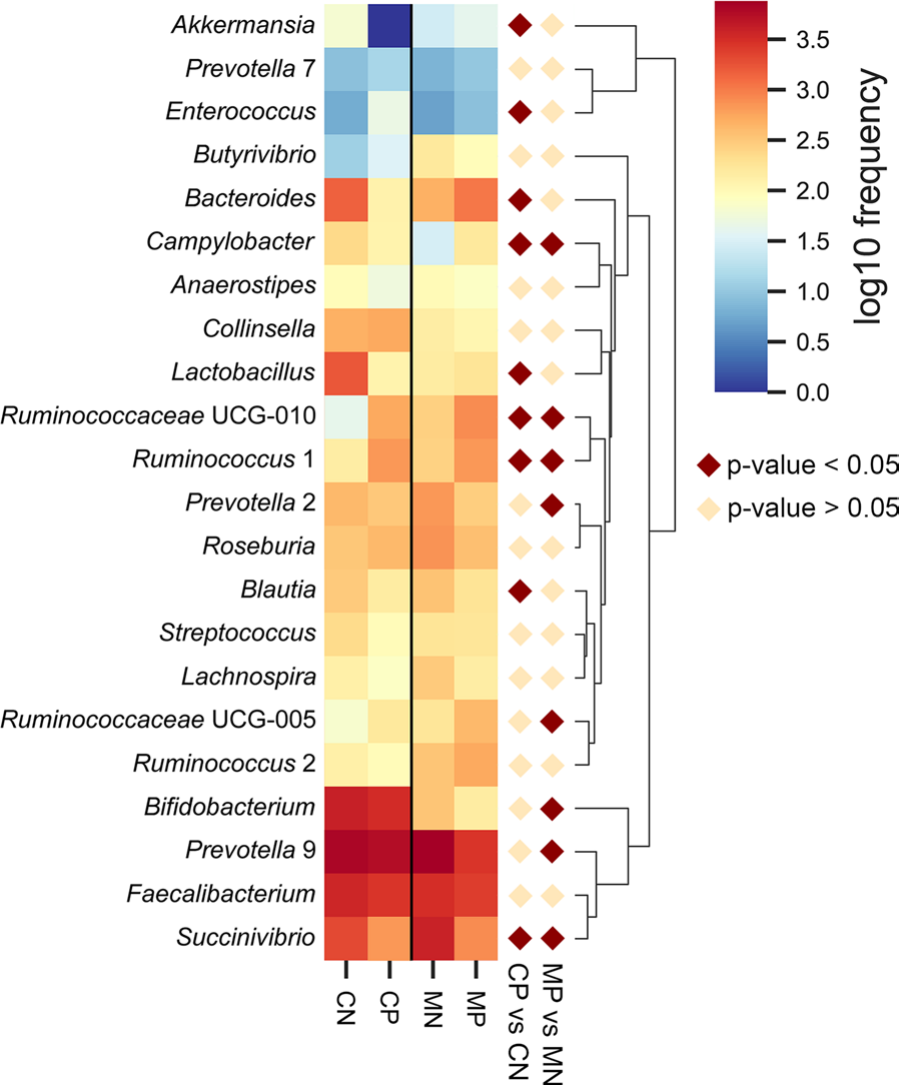
Heatmap plot depicting the normalized abundance of each bacterial genus in mother and children individuals. *P*-values reported were obtained using a Student’s *t*-test. MP, mother helminth-positive group; MN, mother helminth-negative group; CP, children helminth-positive group; CN, children helminth-negative group

We further analyzed the microbial composition at the genus level. The 20 most abundant bacterial genera for each group are shown in Fig. 3. Detailed data of the abundances of each genus are shown in Additional file 5: Table S3. A heat map was also constructed to display microbial genera showing the largest differences in relation to helminth infection in mothers and children, respectively. *Succinivibrio* (phylum Proteobacteria) showed significantly lower abundance in both helminth-positive groups, while *Ruminococcus* 1 and *Ruminococcaceae* UCG-010 both belonging to the phylum Firmicutes showed higher abundance in both helminth-positive groups. *Akkermansia* (phylum Verrucomicrobia), *Lactobacillus, Blautia, Ruminococcus* UCG-005 and *Enterococcus* (phylum Firmicutes), *Bifidobacterium* (phylum Actinobacteria), *Bacteroides* and *Prevotella* 2 and 9 (phylum Bacteroidetes) and *Campylobacter* (phylum Proteobacteria) showed different trends between mothers and children (Fig. 4).

**Fig. 5:**
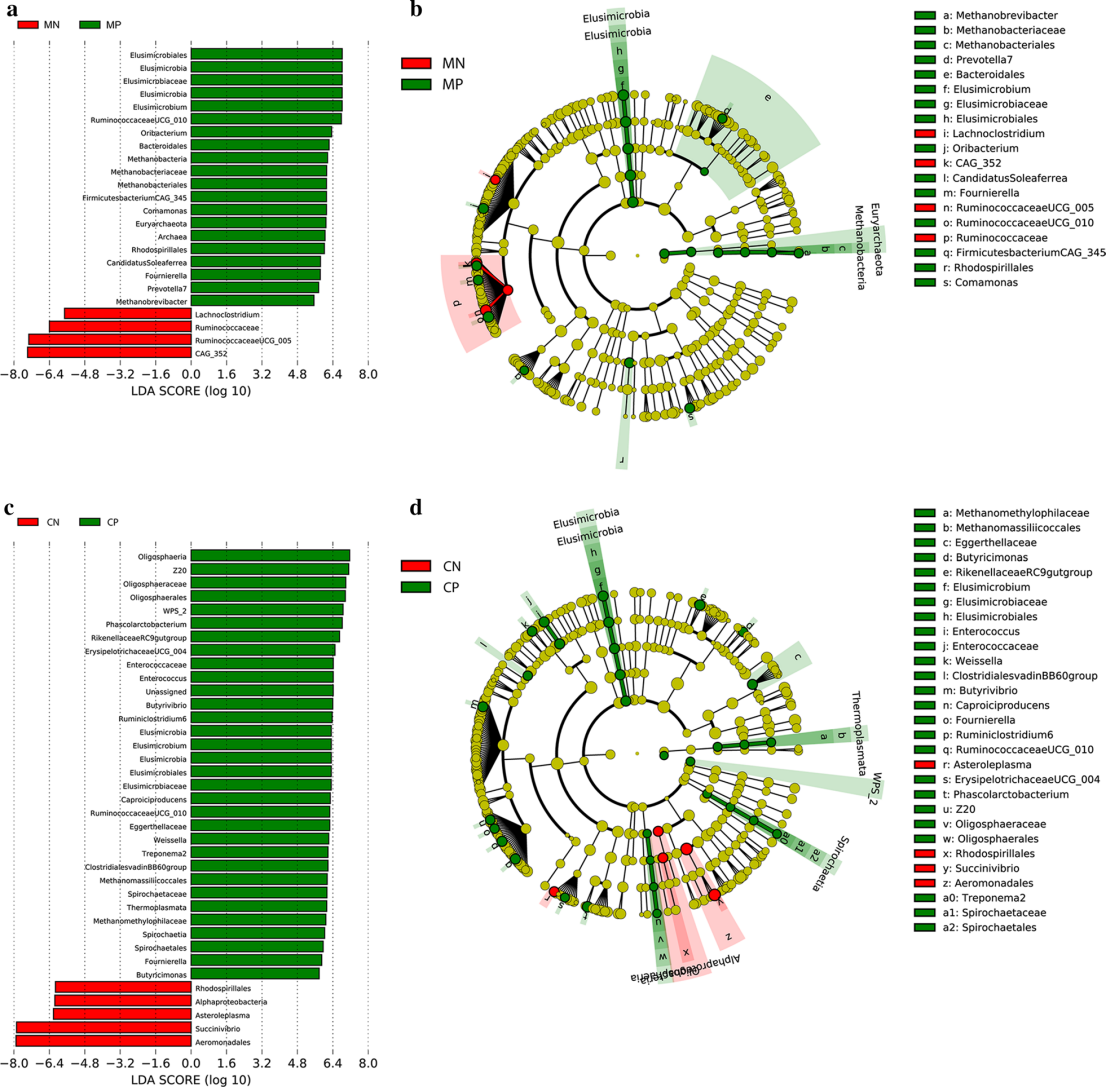
LEfSe histograms [LDA score (log10) cut-off = 3.0] and cladograms displaying differences between helminth-infected (green) and uninfected (red) mothers (**a** and **b**) and children (**c** and **d**). *Abbreviations*: MP, mother helminth-positive group; MN, mother helminth-negative group; CP, children helminth-positive group; CN, children helminth-negative group

We also conducted a LEfSe analysis as an additional approach to identify the significant differences in the abundance of gut microbial populations between groups MN and MP and groups CN and CP separately. The taxonomic cladogram and LDA score (threshold = 3.2) confirmed and enabled the visualization of the significant variations (Fig. 5). The LEfSe analysis showed that compared with the MN group, the abundances of *Ruminococcaceae*, *Ruminococcaceae* UCG-005 and *Lachnoclostridium* were significantly lower in group MP, while *Methanobrevibacter* and *Ruminococcaceae* UCG-010 were significantly higher in group MP. In infected children, *Succinivibrio*, *Asteroleplasma*, *Alphaproteobacteria*, *Rhodospirillales* and *Aeromonadales* were significantly lower with respect to healthy children, while the populations of *Enterococcaceae*, *Ruminococcaceae* UCG-010 and *Enterococcus* were more enriched (Fig. 5).

### Predicted functional KEGG pathways by PICRUSt2

**Fig. 6:**
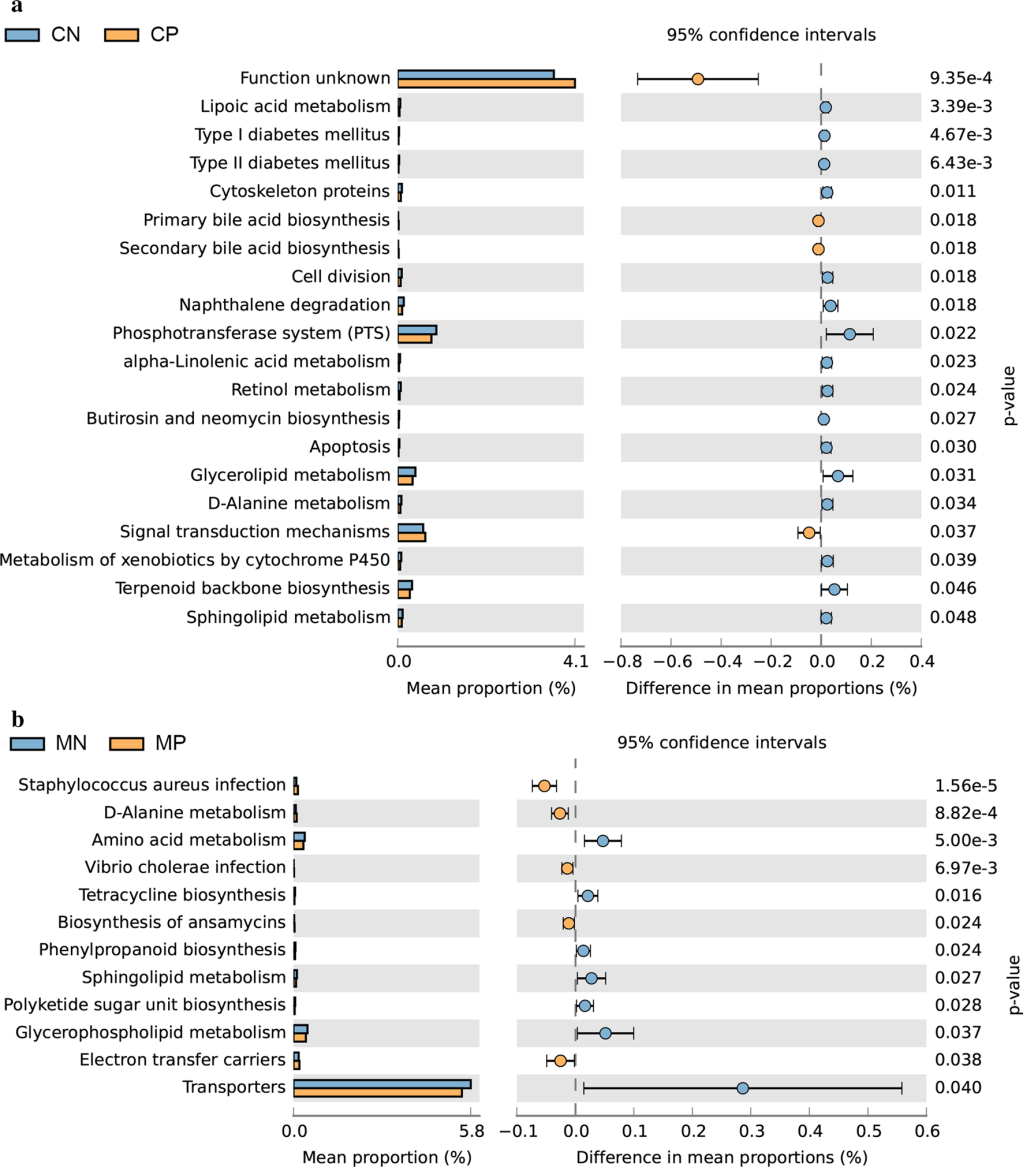
Differences in the abundances of KEGG pathways inferred by PICRUSt2: abundances of KEGG pathways encoded in the gut microbiota between helminth-infected and uninfected children (**a**) and mothers (**b**). *Abbreviations*: MP, mother helminth-positive group; MN, mother helminth-negative group; CP, children helminth-positive group; CN, children helminth-negative group

We observed significant differences in predicted functional abundances between the gut microbiota of helminth-infected and non-infected mothers and children, respectively, by PICRUSt2. Several pathways such as “glycerolipid metabolism” and “sphingolipid metabolism” were less abundant in both infected mothers and children compared to their respective uninfected counterparts (Fig. 6). Infected children showed less abundance in some essential pathways, such as “cytoskeleton proteins,” “cell division” and “apoptosis” compared to non-infected children. Infected mothers showed more abundance in pathways potentially related to pathologies, such as “*Staphylococcus aureus* infection,” “*Vibrio cholerae* infection” and “biosynthesis of ansamycins” compared to non-infected mothers (Fig. 6).

## Discussion

This study showed that *T. trichiura* infection was associated with changes in overall gut microbial composition in both mothers and children. Consistent with the findings of a previous study, Firmicutes, Bacteroidetes, Proteobacteria and Actinobacteria were the most dominant phyla in the human gut microbiota, and Firmicutes was the dominant phylum overall, with the highest abundance in all participants [14]. Alpha diversity describes the richness and evenness of the microbial samples, and beta diversity provides a measure of the distance or dissimilarity between groups of samples [29]. Significant differences in both alpha and beta diversities were found between the helminth-positive and helminth-negative groups in our study, particularly in the mothers’ groups. This finding is different from the previous studies. Jenkins et al. reported no significant changes in alpha diversity (Shannon index) and richness between helminth-positive and helminth-negative groups and significantly greater beta diversity in the helminth-positive group [30]. Additionally, Lee et al. reported that helminth colonization induced no significant changes in the alpha diversity of taxa; only greater richness and enrichment of Paraprevotellaceae were observed [14]. Finally, a comparison of the effects of *T. trichiura* and *A. lumbricoides* colonization on the fecal microbiota showed that only the latter was associated with a disturbed microbiota [31]. While all previous investigations support associations between helminth infection and variations in gut microbial communities, the discrepancies among these studies may be due to many factors, such as endemic areas, the type and level of helminth infections, the nutritional habits of the investigated population and the use of anthelmintic drugs [32].

Although helminth infection appeared to increase gut microbial biodiversity in both mothers and children, the abundances of well-known beneficial bacteria were lower in infected individuals, and other potentially opportunistic pathogenic bacteria showed higher abundances. The significant reductions in *Blautia* and *Bacteroides* in infected children and *Prevotella* 2 and 9 in infected mothers may limit the production of SCFAs with immunoregulatory effects to maintain mucosal homeostasis and to regulate ulcerative colitis [33]. Since SCFAs are precursors of glycerophospholipids and glycerolipids [34], the prediction by PICRUSt2 analysis of reduced metabolism of these lipids in infected mothers and children compared with uninfected mothers and children appears consistent with our hypothesis of a reduction in SCFA production in infected individuals. Moreover, the lower amount of *Succinivibrio,* as supported by both heat map and LEfSe results, in infected children may disrupt carbohydrate digestion because *Succinivibrio* is involved in starch, hemicellulose and xylan degradation, similar to *Prevotella* [35, 36]. Finally, *Campylobacter*, which is potentially pathogenic in humans and typically represented by *C. jejuni* [37], exhibited significantly higher abundance in infected mothers. Although this variation was only validated in the heat map analysis by Student's *t*-test, not supported by the LEfSe algorithm, suggesting the possibility of marginal differences, this variation cannot be ignored since global surveillance of campylobacteriosis has recently been proposed because of the increasing number of pathogenic species found in humans presenting resistance to various antibiotics [38].

The changes in infected mothers and in their children compared with the uninfected groups were similar at both the phylum and genus levels. The possibility that these similarities are due to the maternal relationship in the four infected mother-child pairs should be considered. However, the numbers of shared OTUs at the genus level showed great variability in each infected and uninfected pair, reflecting the peculiar characteristics of the microbiota of children [39]. Therefore, it seems unlikely that the statistically significant changes in both infected groups are due only to maternal relationships.

Although similarities were found between infected mothers and children, some differences were also observed. *Akkermansia* and *Lactobacillus* appeared to be more enriched in group MP than in group MN. However, their abundances were significantly reduced in group CP compared with group CN. *Akkermansia* spp. is considered a beneficial, protective bacterium, as it is a mucin degrader that converts mucin to SCFAs, which may mediate anti-inflammatory effects [40]. Jenkins et al. reported that a significant increase in *A. muciniphila* was present in rural Sri Lankan populations infected with helminths compared with uninfected populations [30], and *T. trichiura* infection dramatically increased the production of mucins in experimental macaques [41]. Therefore, the expansion of *Akkermansia*, whose sole source of energy is mucin, in group MP was expected as a consequence of *T. trichiura* infection, as also previously reported [42], but the significantly lower abundance of *Akkermansia* in group CP compared with CN may suggest concerns for gut protection for infected children. Additionally, in group CP, the significantly lower abundance of *Lactobacillus*, which is commonly used in probiotic products based on its beneficial effects on digestion processes and ability to counteract pathogenic intestinal microbiota and promote host immunomodulation [43, 44], suggests concerns for the gut homeostasis of infected children.

The life cycles of *T. trichiura* and *A. lumbricoides* are different, and *T. trichiura* does not migrate to the pulmonary circulation through the lungs. *T. trichiura* larvae attach to the intestinal villi and develop into adult worms, which then migrate and reside in the cecum and colon. However, after migration and molting, adult *A. lumbricoides* worms colonize the upper small intestine [45]. We hypothesize that the variations in colon bacteria observed in our study are associated with *T. trichiura* infection because of the colonization of same site. *Trichuris* infection could increase the secretion of mucus, which would provide an energy source for adapted microorganisms, resulting in changes in mucosal microbial composition [41, 46, 47]. The potential role of the significant increase in the mucus colonizer *Methanobrevibacter* in group MP and *Ruminococcaceae* UCG-010 in groups MP and CP in this mechanism is of interest for further research using animal models.

The significant increase in *Elusimicrobium* in all infected individuals detected by LEfSe analysis attracted our attention. *Elusimicrobium* is an acetate producer that could maintain intestinal homeostasis [48, 49]. We propose that *Elusimicrobium* is of interest for future studies to test possible therapeutic effects on inflammatory bowel disease (IBD). Since *Lactobacillus* decreased in infected children, follow-up research should also focus on this genus. A previous study demonstrated that oral supplementation with live *Lactobacillus rhamnosus* at a dose of 1 × 10^9^ CFU/day could significantly accelerate larval removal in *T. muris*-resistant C57BL/6 mice [13]. Furthermore, persistent *T. muris* infection notably increases the population of the genus *Lactobacillus* but causes a reduction in the populations of other bacterial species in the gut [50]. The interactions between *T. muris* and *Lactobacillus* may be mutually beneficial rather than causing the microbes to eliminate each other [51]. These findings indicated that some probiotics are friendly to humans and at the same time they may have similar beneficial effects on parasites. Therefore, on the basis of the interplay between *Trichuris* and probiotics, it can be hypothesized that the *Trichuris* therapy, already applied in a few cases [52, 53], may promote growth of “friendly” bacteria to increase gut diversity to treat human IBD.

## Conclusions

Taken together, the results of our study provide a preliminary view of the effects of helminth colonization on the human gut microbiota in mothers and children from Pemba Island. Our data demonstrate that changes in gut microbial composition and structure occur in *T. trichiura*-infected individuals compared with uninfected individuals. Potentially, SCFAs-producing bacteria (*Blautia* and *Bacteroides* in children; *Prevotella* 2 and 9 and *Ruminococcaceae* UCG-005 in mothers) decrease in abundance in infected individuals. Similar decreases were observed in the beneficial bacteria *Lactobacillus* (in children) and *Bifidobacterium* (in mothers) and carbohydrate-degrading *Succinivibrio* in both infected groups. Conversely, potentially pathogenic bacteria, such as *Enterococcus* in infected children and *Campylobacter* in infected mothers, increased in abundance. Our findings could help to improve our current understanding of microbiota profiling in *T. trichiura*-infected individuals, indicate potential roles of key microbiota in the host and contribute to the development of new strategies to control *T. trichiura* infection.

## Supplementary information

**Additional file 1: Material S1.** The questionnaire used in this study for each volunteer.

**Additional file 2: Table S1.** Sample codes of all the 32 pairs of mothers and children and infection burdens of helminth-infected participants.

**Additional file 3: Figure S1.** Rarefaction curves of the OTUs obtained from mother and children individuals.

**Additional file 4: Table S2.** Statistical difference of pairwise comparisons at phylum level. *Abbreviations*: MP, mother helminth-positive group; MN, mother helminth-negative group; CP, children helminth-positive group; CN, children helminth-negative group.

**Additional file 5: Table S3.** Statistical difference of pairwise comparisons at genus level. *Abbreviations*: MP, mother helminth-positive group; MN, mother helminth-negative group; CP, children helminth-positive group; CN, children helminth-negative group.

## Data Availability

Data supporting the conclusions of this article are included within the article and its additional files. All the raw reads were deposited into the NCBI Sequence Read Archive database under the accession number SRP259507, BiopProject accession number PRJNA629760.

## Abbreviations

SCFA: Short-chain fatty acid
STHs: Soil-transmitted helminthiases
NTDs: Neglected tropical diseases
DALYs: Disability-adjusted life years
PHL-IdC: Public Health Laboratory Ivo de Carneri
FLASH: Fast length adjustment of short reads
Qiime: Quantitative insights into microbial ecology
OTU: Operational taxonomic units
PCA: Principal components analysis
LDA: Linear discriminant analysis
LEfSe: Linear discriminant analysis coupled with the effect size
IBD: Inflammatory bowel disease

## References

[1] Palmeirim MS, Hurlimann E, Knopp S, Speich B (2018). Efficacy and safety of co-administered ivermectin plus albendazole for treating soil-transmitted helminths: a systematic review, meta-analysis and individual patient data analysis. Plos Neglected Trop Dis.

[2] Murray CJL, Vos T, Lozano R (2012). Disability-adjusted life years (DALYs) for 291 diseases and injuries in 21 regions, 1990–2010: a systematic analysis for the Global Burden of Disease Study 2010. Lancet.

[3] Owada K, Nielsen M, Lau CL, Clements ACA, Yakob L (2017). Measuring the effect of soil-transmitted helminth infections on cognitive function in children: systematic review and critical appraisal of evidence. Adv Parasitol.

[4] Jourdan PM, Lamberton PHL, Fenwick A, Addiss DG (2018). Soil-transmitted helminth infections. Lancet..

[5] Rapin A, Harris NL (2018). Helminth-bacterial interactions: cause and consequence. Trends Immunol.

[6] Geerts S, Gryseels B (2001). Anthelmintic resistance in human helminths: a review. Tropical Med Int Health.

[7] Truscott JE, Turner HC, Anderson RM (2015). What impact will the achievement of the current World Health Organisation targets for anthelmintic treatment coverage in children have on the intensity of soil transmitted helminth infections?. Parasites Vectors..

[8] Kelly D, Conway S, Aminov R (2005). Commensal gut bacteria: mechanisms of immune modulation. Trends Immunol.

[9] Backhed F, Ley RE, Sonnenburg JL, Peterson DA, Gordon JI (2005). Host-bacterial mutualism in the human intestine. Science.

[10] Oliveira-Sequeira TC, David EB, Ribeiro C, Guimaraes S, Masseno AP (2014). Effect of *Bifidobacterium animalis* on mice infected with *Strongyloides venezuelensis*. Rev Inst Med Trop Sao Paulo.

[11] Reynolds LA, Smith KA, Filbey KJ, Harcus Y, Hewitson JP (2014). Commensal-pathogen interactions in the intestinal tract: *lactobacilli* promote infection with, and are promoted by, helminth parasites. Gut Microbes..

[12] Jiang HY, Zhao N, Zhang QL, Gao JM, Liu LL (2016). Intestinal microbes influence the survival, reproduction and protein profile of *Trichinella spiralis* in vitro. Int J Parasitol.

[13] McClemens J, Kim JJ, Wang HQ, Mao YK, Collins M (2013). *Lactobacillus rhamnosus* ingestion promotes innate host defense in an enteric parasitic infection. Clin Vaccine Immunol.

[14] Lee SC, Tang MS, Lim YAL, Choy SH, Kurtz ZD (2014). Helminth colonization is associated with increased diversity of the gut microbiota. PLoS Neglected Trop Dis..

[15] Jenkins TP, Formenti F, Castro C, Piubelli C, Perandin F (2018). A comprehensive analysis of the faecal microbiome and metabolome of *Strongyloides stercoralis* infected volunteers from a non-endemic area. Sci Rep.

[16] Giacomin P, Zakrzewski M, Croese J, Su X, Sotillo J (2015). Experimental hookworm infection and escalating gluten challenges are associated with increased microbial richness in celiac subjects. Sci Rep.

[17] Giacomin P, Zakrzewski M, Jenkins TP, Su X, Al-Hallaf R (2016). Changes in duodenal tissue-associated microbiota following hookworm infection and consecutive gluten challenges in humans with coeliac disease. Sci Rep.

[18] Peachey LE, Jenkins TP, Cantacessi C (2017). This gut ain't big enough for both of us. Or Is It? Helminth-microbiota interactions in veterinary species. Trends Parasitol..

[19] Zaiss MM, Rapin A, Lebon L, Dubey LK, Mosconi I (2015). The intestinal microbiota contributes to the ability of helminths to modulate allergic inflammation. Immunity.

[20] Giacomin P, Croese J, Krause L, Loukas A, Cantacessi C (2015). Suppression of inflammation by helminths: a role for the gut microbiota?. Philos Trans R Soc London Series B Biol Sci..

[21] Reda AA (2018). Probiotics for the control of helminth zoonosis. J Veterinary Med.

[22] Cringoli G, Maurelli MP, Levecke B, Bosco A, Vercruysse J (2017). The Mini-FLOTAC technique for the diagnosis of helminth and protozoan infections in humans and animals. Nat Protoc.

[23] Barda B, Zepherine H, Rinaldi L, Cringoli G, Burioni R (2013). Mini-FLOTAC and Kato-Katz: helminth eggs watching on the shore of Lake Victoria. Parasites Vectors..

[24] Herlemann DPR, Labrenz M, Jurgens K, Bertilsson S, Waniek JJ (2011). Transitions in bacterial communities along the 2000 km salinity gradient of the Baltic Sea. ISME J.

[25] Martin M (2011). Cutadapt removes adapter sequences from high-throughput sequencing reads. EMBnet J.

[26] Bolyen E, Rideout JR, Dillon MR, Bokulich NA, Abnet CC (2019). Reproducible, interactive, scalable and extensible microbiome data science using QIIME 2. Nat Biotechnol.

[27] Segata N, Izard J, Waldron L, Gevers D, Miropolsky L (2011). Metagenomic biomarker discovery and explanation. Genome Biol.

[28] Douglas GM, Maffei VJ, Zaneveld JR, Yurgel SN, Brown JR (2020). PICRUSt2 for prediction of metagenome functions. Nat Biotechnol.

[29] Anderson MJ, Ellingsen KE, Mcardle BH (2010). Multivariate dispersion as a measure of beta diversity. Ecol Lett.

[30] Jenkins TP, Rathnayaka Y, Perera PK, Peachey LE, Nolan MJ (2017). Infections by human gastrointestinal helminths are associated with changes in faecal microbiota diversity and composition. PLoS ONE.

[31] Cooper P, Walker AW, Reyes J, Chico M, Salter SJ (2013). Patent human infections with the whipworm, *Trichuris trichiura*, are not associated with alterations in the faecal microbiota. PLoS ONE.

[32] Cortés A, Peachey LE, Jenkins TP, Scotti R, Cantacessi C (2019). Helminths and microbes within the vertebrate gut—not all studies are created equal. Parasitology.

[33] Zhang D, Liu H, Wang S, Zhang W, Wang J (2019). Fecal microbiota and its correlation with fatty acids and free amino acids metabolism in piglets after a *Lactobacillus* strain oral administration. Front Microbiol.

[34] Kindt A, Liebisch G, Clavel T, Haller D, Hormannsperger G (2018). The gut microbiota promotes hepatic fatty acid desaturation and elongation in mice. Nat Commun.

[35] Schnorr SL, Candela M, Rampelli S, Centanni M, Consolandi C (2014). Gut microbiome of the Hadza hunter-gatherers. Nat Commun.

[36] Obregon-Tito AJ, Tito RY, Metcalf J, Sankaranarayanan K, Clemente JC (2015). Subsistence strategies in traditional societies distinguish gut microbiomes. Nat Commun.

[37] Cover TL, Blaser MJ (1989). The pathobiology of *Campylobacter* infections in humans. Annu Rev Med.

[38] Facciola A, Riso R, Avventuroso E, Visalli G, Delia SA (2017). *Campylobacter*: from microbiology to prevention. J Preventive Med Hygiene..

[39] Derrien M, Alvarez AS, de Vos WM (2019). The gut microbiota in the first decade of life. Trends Microbiol.

[40] Derrien M, Vaughan EE, Plugge CM, de Vos WM (2004). Akkermansia muciniphila gen. nov., sp. nov., a human intestinal mucin-degrading bacterium. Int J Syst Evol Microbiol.

[41] Broadhurst MJ, Ardeshir A, Kanwar B, Mirpuri J, Gundra UM (2012). Therapeutic helminth infection of macaques with idiopathic chronic diarrhea alters the inflammatory signature and mucosal microbiota of the colon. Plos Pathog..

[42] Derrien M, Collado MC, Ben-Amor K, Salminen S, de Vos WM (2008). The mucin degrader *Akkermansia muciniphila* is an abundant resident of the human intestinal tract. Appl Environ Microb..

[43] Markowiak P, Slizewska K (2017). Effects of probiotics, prebiotics, and synbiotics on human health. Nutrients..

[44] Dicks LM, Botes M (2010). Probiotic lactic acid bacteria in the gastro-intestinal tract: health benefits, safety and mode of action. Beneficial Microbes..

[45] Holland C (2010). Gastrointestinal Nematodes *Ascaris*, Hookworm, *Trichuris*, and *Enterobius* Topley and Wilson's Microbiology and Microbial Infections.

[46] Hasnain SZ, Dawson PA, Lourie R, Hutson P, Tong H (2017). Immune-driven alterations in mucin sulphation is an important mediator of *Trichuris muris* helminth expulsion. Plos Pathog..

[47] Derrien M, van Passel MW, van de Bovenkamp JH, Schipper RG, de Vos WM (2010). Mucin-bacterial interactions in the human oral cavity and digestive tract. Gut microbes..

[48] Geissinger O, Herlemann D, Mörschel E, Maier U, Brune A (2009). The ultramicrobacterium "Elusimicrobium minutum" gen. nov., sp. Nov., the first cultivated representative of the termite group 1 phylum. Appl Environ Microb..

[49] Parada Venegas D, De la Fuente MK, Landskron G, González MJ, Quera R (2019). Short chain fatty acids (SCFAs)-mediated gut epithelial and immune regulation and its relevance for inflammatory bowel diseases. Front Immunol.

[50] Holm JB, Sorobetea D, Kiilerich P, Ramayo-Caldas Y, Estelle J (2015). Chronic *Trichuris muris* infection decreases diversity of the intestinal microbiota and concomitantly increases the abundance of *Lactobacilli*. PLoS ONE.

[51] Zaiss MM, Harris NL (2016). Interactions between the intestinal microbiome and helminth parasites. Parasite Immunol.

[52] Huang X, Zeng LR, Chen FS, Zhu JP, Zhu MH (2018). *Trichuris suis* ova therapy in inflammatory bowel disease: a meta-analysis. Medicine..

[53] Summers RW, Elliott DE, Urban JF, Thompson R, Weinstock JV (2005). *Trichuris suis* therapy in Crohn's disease. Gut.

