# Supplementary material for: Dissection of the gut microbiota in mothers and children with chronic Trichuris trichiura infection in Pemba Island, Tanzania
Source: Parasit Vectors. 2021 Jan 19;14:62. doi: 10.1186/s13071-021-04580-1 (PMC7814639; doi:10.1186/s13071-021-04580-1)
Supplement: Supplementary file 1 — Additional file 1: Material S1. The questionnaire used in this study for each volunteer. [file 13071_2021_4580_MOESM1_ESM.pdf]

QUESTIONNAIRE

Fill out the questionnaire carefully (with the help of the nurse) because it is a fundamental part of your contribution.
It takes about 5 minutes to complete it.
If you have any doubts, do not hesitate to contact us.

We would ask you a contact/address to send you the parasitological results of your sample which can assist in the provision of treatment and care.

Thanks from the staff.

Mother

Sample code:

Parameters:

Age	Height (cm)	Weight (Kg)
Abdominal circumference (cm)	WHR	BMI

Personal (with the help of the nurse):

1. Describe your typical day, what do you usually do?
2. Which sheias are you from? Which village?
3. How did you come here? Did you take dala dala or other transports? How do you usually go to the town?
4. How old is your first born? And how old is your last born?
5. Is this your child?
6. How long did you breastfeed your last born? Or you still breastfeeding?
7. For what concern you, do you remember if have you been breastfeeding?
8. If yes, how many months?
9. How many people do you live with?
10. Do you have animals inside the house? And outside? Which kind of animals?
11. Where do you take the water for washing yourself and your baby?
12. Where do you take the water for washing dishes and clothes?
13. Do you have toilet or latrine at home?
14. Are you pregnant? yes☐ no☐
15. If yes, which month?

Feces:

1. Do you usually suffer from diarrhea? For example, did you suffer from it in the previous month? How many times?
2. Are you suffering from diarrhea now?
3. Normal appearance of stool?

Nutritional Habits:

1. Describe your typical day, what do you eat? What did you eat yesterday? And this week?
2. Have you done pica during pregnancy?
3. Do you usually buy your food?
4. Do you wash your food? With which water?
5. Do you use cutlery for eat? Or by hands?
6. How do you consume your food? Raw or cooked?
7. Do you drink during the meals? And outside the meal? How many times? (in terms of glasses, bottle, ecc.)
8. Do you buy water in the bottle? If not, what water do you use?
9. Do you prepare your food?

Personal health (with the help of the nurse):

1. In the last year do you suffer from:

HIV	Diabetes	Malaria	Anything else

2. Do you have any vaccination?
3. If yes, specify which ……………………………………………
4. Do you suffer from any of the following symptoms at the time of collection?

Diarrhea	Soft stool	Skin rash	Confusion of mind	Dizziness	Headache	Stomach pains
Anal fissures	Constipation	Meteorism	Insomnia	Muscle pain	Temperature	Anything else

Medications:

1. Do you take any medicines? yes☐ no☐
2. If yes, which medicines do you take? ……………………………………………

Child

Sample code:

Parameters:

Sex M☐ F☐	Age	Height (cm)	Weight (Kg)
Weight-for-height Z-score (WHZ)	Weight-for-age Z-score (WAZ)	Height-for-age Z-score (HAZ)	BMI
Birth weight	Born prematurely yes☐ no☐	Body temperature	

Personal:

1. He/she has been breastfeeding initially? Still breastfeeding? How long he/she has been breastfeeding?
2. Did he/she take any supplementation other than milk? yes☐ no☐
3. He/she has start to walk?

Feces:

1. Do he/she usually suffer from diarrhea? For example, did he/she suffer from it in the previous months? How many times?
2. Is he/she suffering from diarrhea now?
3. Normal appearance of stool?

Nutritional Habits:

1. Ask to the mother to describe what he/she usually eat
2. If he/she eat fruits (fruits from the tree or bought at the market?)
3. Did he/she eat fruits or other during the breastfeeding?
4. Ask to the mother about the weaning
5. He/she usually eat candy or sugar? Or sweetened drink?
6. How does he/she consume food? Raw or cooked?
7. Does he/she drink during the meals? And outside the meal? How many times? (in terms of glasses, bottle, ecc.)
10. Does he/she drink water in the bottle? If not, what water does he/she use?

Health:

1. Did he/she received any vaccination?
2. If yes, specify which ……………………………………………
3. Does he/she suffer from any of the following symptoms at the time of collection?

Blood in the stool	Diarrhea	Soft stool
Skin rash	Stomach pains	Constipation
Insomnia	Fever	Anything else

Medications:

1. Does he/she take any medicines? yes☐ no☐
2. If yes, which medicines does he/she take? ……………………………………………

INFORMED CONSENT TO PARTICIPATE IN A RESEARCH STUDY

Title of Project: **Analysis of intestinal microbiota of mother and children of Pemba and study of the associations among nutritional habits, gut microbiota, and parasites**

1. Introduction

From the time of the delivery, the human body starts to be colonized by many microbes in different sites. All of the different kinds of microbes that live on and inside us, combined, are called the "human microbiome." Those of the gut are called "gut microbiome".
Most of these microbes are commensal, so they are not harmful but help to digest food and to maintain immune systems. Some of them have been so far considered essentially infective or parasites, but it is now clear that their interaction with the commensal microbes and with the body cells deserves attention and may have not only negative effects.
The gut microbiota is considered an essential component of the human life.
In this proposed study we hope to contribute to the knowledge related to the gut microbiome. In particular, we are interested in understanding variations of the prevalence of microbial species in relation to the diet, presence of other microorganisms that can have effects on health and look for potentially beneficial microbes associated to better nutritional status, anthropometric parameters, and low parasite levels.
The samples that you provide for this study may be used for further investigations to better understand relationship of microbiota and nutritional deficiencies.
You will get a feedback of the parasitological results of your sample which can assist you in the provision of treatment and care.

2. What are you being asked to do?

If you agree to participate, you will be asked to sign this form. After that we will ask you few questions in relation to your family, nutrition and general health which may last for about 10 minutes. In addition, we will provide stool containers for you and your child and asked to provide us with a small amount of stool sample from you and your child in the next day. We will also record vital signs (temperature, height and weight) for you and your child.

We will ask you and your child to avoid using antibiotics that could alter the microbes in your body before the sampling. If you or your child/children use any medications you should declare the product you used.

3. What will happen to your/your child's sample?

The samples will be sent to the PHL-IdC where they will be assessed for the presence of protozoa and helminths using mini-Flotac technique. In addition, DNA will be extracted from the samples and will be sent to Italy for further analysis.

4. What are the benefits and the risk for participating?

It will not cost you anything to take part in this project other than your time that you will spend in answering the accompanied questions and submission of samples. However, we will reimburse your transport fare (Tsh 5,000 equivalent to 2€). In addition we will give you feedback of the parasitological analysis as benefit.

The research involves no more than minimal risks, both for you and your child/children:

- **Interviews/Questionnaires:** Some questions may make you uncomfortable; however, you may refuse to answer any question for any reason.

5. What other options are there?
Taking part in this research study is voluntary. You may choose not to take part in this research study or you may withdraw your consent at any time. Please note, once information from the study of your samples has been placed in the databases, you will not be able to withdraw that information, only to stop any additional information from being put in the databases.
By signing this consent form, you have authorized the use of your sample for this research.

6. What about privacy and confidentiality?
The samples that you and your child give us will not be labeled with your name or any other traditional identifying information (for example, address, telephone number, Social Security number). Your samples will be labeled only with code numbers (de-identified), and the link between these codes and your identity will be stored in a locked file and password protected computer. Only a very small number of authorized people involved in this project will have access to the link between sample code numbers and your identity.

7. Whom do you call if you have Questions or Problems?
Please contact the researcher listed below to:
• Obtain more information about the study
• Ask a question about the study procedures

Principal Investigators: Dr. Cristina Miceli, Dr. Shaali Ame and Mr. Said Mohammed
Mailing Address: cristina.miceli@unicam.it; shaaliame@yahoo.com; saidmali2003@yahoo.com
If you wish to talk to someone or have questions or concerns about your rights as a research subject, call Dr. Shaali Ame (0777432094) and Mr. Said Mohammed (0777416867).

8. You will be given a signed copy of this consent form for your records. I have read this consent form. I have been given the chance to ask questions and all my questions have been clearly answered. I agree to participate in this research described above. titled: "**Analysis of intestinal microbiota of mother and children of Pemba and study of the associations among nutritional habits, gut microbiota, and parasites**".

Signature: ______________________________

Printed Name: ___________________________ Date of Signature: ________________

Principal Investigator (or designee)
I have given this research participant information about this study that I believe is accurate and complete. The participant has indicated that he or she understands the nature of the study and the risks and benefits of participating.
Signature: ______________________________ Title: ___________________________
Printed Name: ___________________________ Date of Signature: ________________
