# Supplementary material for: Dissection of the gut microbiota in mothers and children with chronic Trichuris trichiura infection in Pemba Island, Tanzania
Source: Parasit Vectors. 2021 Jan 19;14:62. doi: 10.1186/s13071-021-04580-1 (PMC7814639; doi:10.1186/s13071-021-04580-1)
Supplement: Supplementary file 2 — Additional file 2: Table S1. Sample codes of all the 32 pairs of mothers and children and infection burdens of helminth-infected participants. [file 13071_2021_4580_MOESM2_ESM.docx]

Table S1: Pairs of mothers (M code) and children (C code) involved in the study and the infection burdens of helminth-infected participants.

| Sample code | *T. trichiura* (eggs/gram of faeces^[[1]](#footnote-1)^) | *Ascaris* (eggs/gram of faeces) | Sample code | *T. trichiura* (eggs/gram of faeces^[[2]](#footnote-2)^) | *Ascaris* (eggs/gram of faeces) | Habited village |
| --- | --- | --- | --- | --- | --- | --- |
| M01 | 10 | 10 | C31 | 0 | 0 | Vitongoji |
| M02 | 0 | 0 | C32 | 20 | 0 | Vitongoji |
| M04 | 200 | 200 |  |  |  | Vitongoji |
| M05 | 20 | 0 | C35 | 100 | 0 | Vitongoji |
| M06 | 20 | 0 | C36 | 0 | 0 | Vitongoji |
| M07 | 0 | 0 | C37 | 0 | 0 | Vitongoji |
| M08 | 0 | 0 | C38 | 0 | 0 | Vitongoji |
| M09 | 450 | 0 | C39 | 0 | 0 | Vitongoji |
| M10 | 0 | 0 | C40 | 0 | 0 | Vitongoji |
| M11 | 20 | 0 | C41 | 30 | 0 | Vitongoji |
| M12 | 0 | 0 | C42 | 0 | 0 | Gombani |
| M13 | 0 | 0 | C43 | 0 | 0 | Gombani |
| M14 | 0 | 0 |  |  |  | Gombani |
| M16 | 0 | 0 |  |  |  | Gombani |
| M17 | 160 | 0 | C47 | 80 | 0 | Gombani |
| M18 | 100 | 0 | C48 | 0 | 0 | Gombani |
| M19 | 0 | 0 | C49 | 0 | 0 | Gombani |
|  |  |  | C50 | 0 | 0 | Gombani |
| M22 | 0 | 0 | C52 | 0 | 0 | Gombani |
| M23 | 700 | 0 | C53 | 250 | 750 | Gombani |
| M25 | 0 | 0 | C55 | 0 | 0 | Chake Chake |
| M26 | 0 | 0 | C56 | 0 | 0 | Chake Chake |
| M28 | 0 |  | C58 | 0 | 0 | Chake Chake |
| M30 | 150 | 0 | C60 | 0 | 0 | Chake Chake |
| M61 | 70 | 0 | C81 | 0 | 0 | Chake Chake |
|  |  |  | C82 | 0 | 0 | Chake Chake |
| M63 | 0 | 0 |  |  |  | Chake Chake |
| M64 | 0 | 0 | C84 | 0 | 0 | Chake Chake |
| M65 | 0 | 0 |  |  |  | Chake Chake |
|  |  |  | C86 | 0 | 0 | Chake Chake |
| M67 | 0 | 0 | C87 | 70 | 0 | Chake Chake |
| M69 | 0 | 0 | C89 | 0 | 0 | Chake Chake |

1. The Mini–FLOTAC technique was utilized for the parasitological analysis. [↑](#footnote-ref-1)
2. The Mini–FLOTAC technique was utilized for the parasitological analysis. [↑](#footnote-ref-2)
