# Supplementary figures and images for: Dissection of the gut microbiota in mothers and children with chronic Trichuris trichiura infection in Pemba Island, Tanzania
Source: Parasit Vectors. 2021 Jan 19;14:62. doi: 10.1186/s13071-021-04580-1 (PMC7814639; doi:10.1186/s13071-021-04580-1)

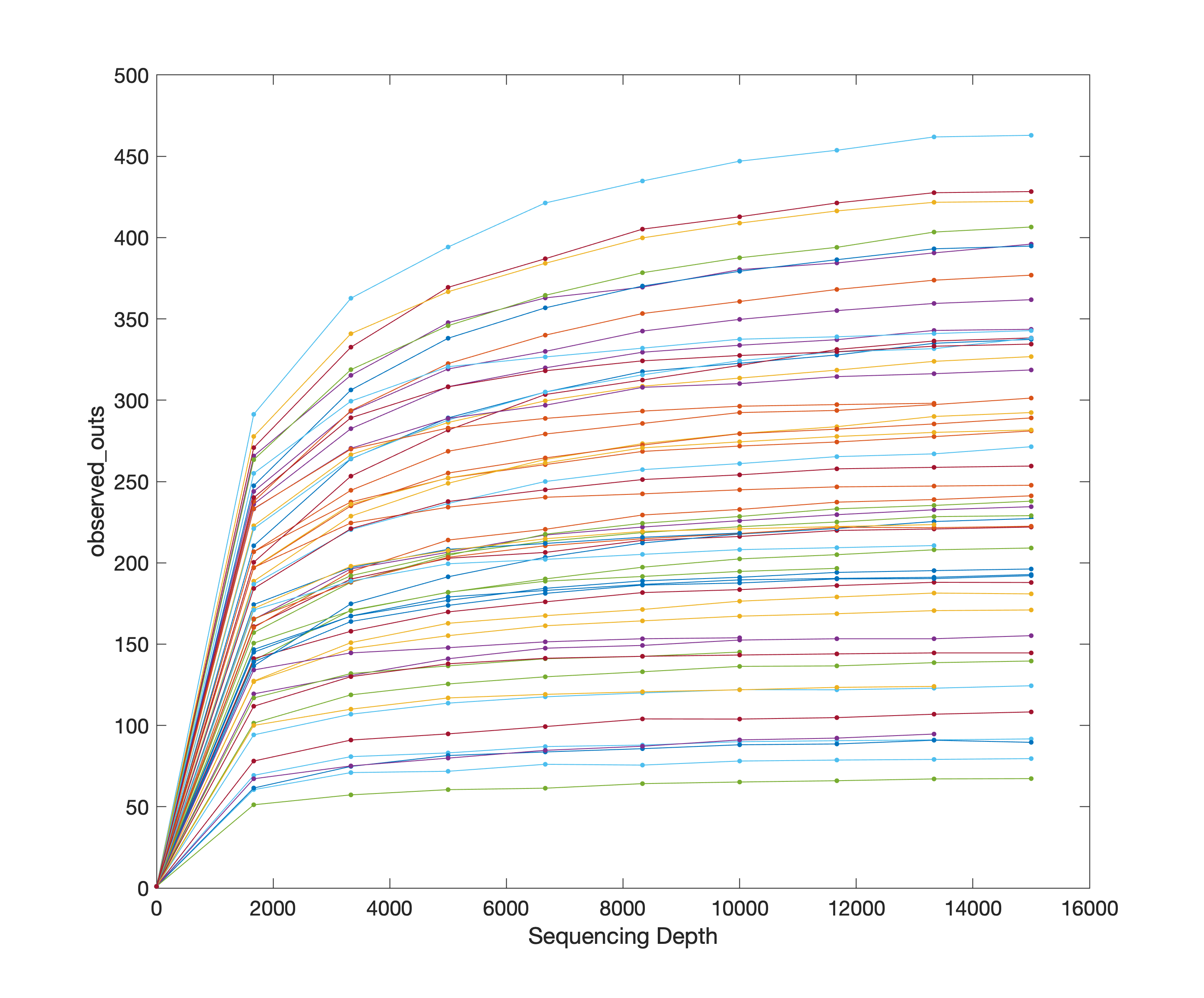

Supplement: Supplementary file 3 — Additional file 3: Figure S1. Rarefaction curves of the OTUs obtained from mother and children individuals. [file 13071_2021_4580_MOESM3_ESM.tif]
