# Supplementary material for: Dissection of the gut microbiota in mothers and children with chronic Trichuris trichiura infection in Pemba Island, Tanzania
Source: Parasit Vectors. 2021 Jan 19;14:62. doi: 10.1186/s13071-021-04580-1 (PMC7814639; doi:10.1186/s13071-021-04580-1)
Supplement: Supplementary file 4 — Additional file 4: Table S2. Statistical difference of pairwise comparisons at phylum level. Abbreviations: MP, mother helminth-positive group; MN, mother helminth-negative group; CP, children helminth-positive group; CN, children helminth-negative group. [file 13071_2021_4580_MOESM4_ESM.docx]

| Phylum | MN |  | MP |  |  | CN |  | CP |  |  |
| --- | --- | --- | --- | --- | --- | --- | --- | --- | --- | --- |
| ID | % | err% | % | err% | p-value < 0.05 | % | err% | % | err% | p-value < 0.05 |
| Euryarchaeota | 0.07 | 0.04 | 0.67 | 0.23 | YES | 0.03 | 0.00 | 0.49 | 0.16 | YES |
| Actinobacteria | 1.79 | 0.20 | 1.38 | 0.15 | YES | 15.55 | 3.51 | 15.61 | 9.85 |  |
| Bacteroidetes | 31.12 | 3.76 | 22.31 | 3.91 | YES | 30.65 | 3.28 | 29.44 | 9.27 |  |
| Cyanobacteria | 0.63 | 0.22 | 0.57 | 0.31 |  | 0.54 | 0.31 | 0.48 | 0.28 |  |
| Elusimicrobia | 0.45 | 0.51 | 1.45 | 1.09 |  | 0.07 | 0.57 | 0.74 | 0.25 |  |
| Epsilonbacteraeota | 0.09 | 0.22 | 0.64 | 1.34 |  | 0.94 | 0.57 | 0.44 | 0.27 |  |
| Firmicutes | 46.90 | 3.58 | 57.46 | 5.24 | YES | 41.42 | 3.87 | 42.28 | 8.22 |  |
| Fusobacteria | 0.10 | 0.36 | 0.00 | 0.00 |  | 0.60 | 0.70 | 0.33 | 0.26 |  |
| Lentisphaerae | 0.04 | 0.04 | 0.13 | 0.09 |  | 0.01 | 0.01 | 0.07 | 0.02 | YES |
| Proteobacteria | 17.73 | 1.89 | 13.78 | 1.41 | YES | 9.72 | 2.80 | 9.44 | 3.07 |  |
| Spirochaetes | 0.57 | 1.29 | 0.33 | 0.37 |  | 0.03 | 0.03 | 0.13 | 0.10 |  |
| Tenericutes | 0.27 | 0.18 | 0.89 | 0.43 | YES | 0.11 | 0.03 | 0.09 | 0.06 |  |
| Verrucomicrobia | 0.09 | 0.05 | 0.17 | 0.29 |  | 0.20 | 0.21 | 0.00 | 0.00 |  |
| WPS-2 | 0.00 | 0.00 | 0.00 | 0.00 |  | 0.00 | 0.00 | 0.30 | 0.81 |  |

Table S2: Statistical difference of pairwise comparisons at phylum level.
