# Supplementary material for: Dissection of the gut microbiota in mothers and children with chronic Trichuris trichiura infection in Pemba Island, Tanzania
Source: Parasit Vectors. 2021 Jan 19;14:62. doi: 10.1186/s13071-021-04580-1 (PMC7814639; doi:10.1186/s13071-021-04580-1)
Supplement: Supplementary file 5 — Additional file 5: Table S3. Statistical difference of pairwise comparisons at genus level. Abbreviations: MP, mother helminth-positive group; MN, mother helminth-negative group; CP, children helminth-positive group; CN, children helminth-negative group. [file 13071_2021_4580_MOESM5_ESM.docx]

Table S3: Statistical difference of pairwise comparisons at genus level.

| The 80 most abundant bacterial genera | MN |  | MP |  |  | CN |  | CP |  |  |
| --- | --- | --- | --- | --- | --- | --- | --- | --- | --- | --- |
| ID | % | err% | % | err% | p-value < 0.05 | % | err% | % | err% | p-value < 0.05 |
| D_0__Bacteria;D_1__Bacteroidetes;D_2__Bacteroidia;D_3__Bacteroidales;D_4__Prevotellaceae;D_5__Prevotella 9 | 22.99 | 2.15 | 10.81 | 2.89 | YES | 21.02 | 2.13 | 20.67 | 6.50 |  |
| D_0__Bacteria;D_1__Proteobacteria;D_2__Gammaproteobacteria;D_3__Aeromonadales;D_4__Succinivibrionaceae;D_5__Succinivibrio | 12.02 | 1.27 | 8.06 | 1.90 | YES | 6.77 | 1.50 | 4.43 | 0.62 | YES |
| D_0__Bacteria;D_1__Firmicutes;D_2__Clostridia;D_3__Clostridiales;D_4__Ruminococcaceae;D_5__Faecalibacterium | 9.90 | 0.78 | 9.40 | 1.27 |  | 11.68 | 1.04 | 10.19 | 0.77 |  |
| D_0__Bacteria;D_1__Firmicutes;D_2__Clostridia;D_3__Clostridiales;D_4__Lachnospiraceae;D_5__Agathobacter | 2.93 | 0.41 | 2.84 | 0.55 |  | 1.42 | 0.58 | 0.48 | 0.39 |  |
| D_0__Bacteria;D_1__Firmicutes;D_2__Clostridia;D_3__Clostridiales;D_4__Ruminococcaceae;D_5__Ruminococcaceae UCG-002 | 2.55 | 0.79 | 4.29 | 0.63 | YES | 0.97 | 0.43 | 4.46 | 3.14 |  |
| D_0__Bacteria;D_1__Firmicutes;D_2__Clostridia;D_3__Clostridiales;D_4__Lachnospiraceae;D_5__Roseburia | 2.30 | 0.54 | 1.42 | 0.41 |  | 1.05 | 0.24 | 1.51 | 0.45 |  |
| D_0__Bacteria;D_1__Bacteroidetes;D_2__Bacteroidia;D_3__Bacteroidales;D_4__Prevotellaceae;D_5__Prevotella 2 | 2.13 | 0.45 | 1.07 | 0.53 | YES | 1.34 | 0.69 | 1.15 | 0.48 |  |
| D_0__Bacteria;D_1__Bacteroidetes;D_2__Bacteroidia;D_3__Bacteroidales;D_4__Prevotellaceae;D_5__Alloprevotella | 2.01 | 0.93 | 1.30 | 0.46 |  | 1.23 | 0.77 | 1.86 | 0.96 |  |
| D_0__Bacteria;D_1__Firmicutes;D_2__Clostridia;D_3__Clostridiales;D_4__Lachnospiraceae;__ | 1.90 | 0.13 | 2.11 | 0.14 |  | 1.11 | 0.12 | 1.24 | 0.32 |  |
| D_0__Bacteria;D_1__Proteobacteria;D_2__Gammaproteobacteria;D_3__Betaproteobacteriales;D_4__Burkholderiaceae;D_5__Sutterella | 1.75 | 0.30 | 1.33 | 0.66 |  | 1.35 | 0.17 | 0.96 | 0.44 |  |
| D_0__Bacteria;D_1__Firmicutes;D_2__Clostridia;D_3__Clostridiales;D_4__Lachnospiraceae;D_5__Lachnospiraceae NK4A136 group | 1.65 | 0.19 | 1.34 | 0.34 |  | 0.78 | 0.21 | 2.17 | 1.20 |  |
| D_0__Bacteria;D_1__Firmicutes;D_2__Clostridia;D_3__Clostridiales;D_4__Ruminococcaceae;D_5__Ruminococcaceae UCG-014 | 1.55 | 0.36 | 2.98 | 0.54 | YES | 0.91 | 0.49 | 1.04 | 0.25 |  |
| D_0__Bacteria;D_1__Firmicutes;D_2__Negativicutes;D_3__Selenomonadales;D_4__Veillonellaceae;D_5__Dialister | 1.54 | 0.22 | 0.94 | 0.22 | YES | 1.11 | 0.23 | 0.75 | 0.34 |  |
| D_0__Bacteria;D_1__Bacteroidetes;D_2__Bacteroidia;D_3__Bacteroidales;D_4__Bacteroidaceae;D_5__Bacteroides | 1.49 | 0.47 | 4.02 | 3.48 |  | 4.68 | 1.03 | 0.44 | 0.21 | YES |
| D_0__Bacteria;D_1__Firmicutes;D_2__Clostridia;D_3__Clostridiales;D_4__Ruminococcaceae;D_5__[Eubacterium] coprostanoligenes group | 1.44 | 0.24 | 2.15 | 0.36 | YES | 0.81 | 0.15 | 1.34 | 0.36 | YES |
| D_0__Bacteria;D_1__Firmicutes;D_2__Clostridia;D_3__Clostridiales;D_4__Lachnospiraceae;D_5__[Eubacterium] eligens group | 1.31 | 0.19 | 0.76 | 0.20 | YES | 0.75 | 0.24 | 1.23 | 0.43 |  |
| D_0__Bacteria;D_1__Firmicutes;D_2__Clostridia;D_3__Clostridiales;D_4__Lachnospiraceae;D_5__Blautia | 1.07 | 0.17 | 0.71 | 0.20 |  | 0.97 | 0.13 | 0.51 | 0.11 | YES |
| D_0__Bacteria;D_1__Actinobacteria;D_2__Actinobacteria;D_3__Bifidobacteriales;D_4__Bifidobacteriaceae;D_5__Bifidobacterium | 1.04 | 0.20 | 0.55 | 0.20 | YES | 12.74 | 1.86 | 11.61 | 6.39 |  |
| D_0__Bacteria;D_1__Firmicutes;D_2__Clostridia;D_3__Clostridiales;D_4__Ruminococcaceae;D_5__Ruminococcus 2 | 1.04 | 0.35 | 1.99 | 0.66 |  | 0.43 | 0.07 | 0.34 | 0.11 |  |
| D_0__Bacteria;D_1__Firmicutes;D_2__Clostridia;D_3__Clostridiales;D_4__Lachnospiraceae;D_5__Lachnospira | 0.94 | 0.22 | 0.53 | 0.25 |  | 0.42 | 0.09 | 0.28 | 0.10 |  |
| D_0__Bacteria;D_1__Firmicutes;D_2__Clostridia;D_3__Clostridiales;D_4__Ruminococcaceae;D_5__Subdoligranulum | 0.88 | 0.07 | 1.11 | 0.12 | YES | 0.95 | 0.14 | 0.94 | 0.11 |  |
| D_0__Bacteria;D_1__Proteobacteria;D_2__Alphaproteobacteria;D_3__Rhodospirillales;D_4__uncultured;D_5__uncultured bacterium | 0.84 | 2.50 | 0.64 | 0.31 |  | 0.00 | 0.00 | 0.10 | 0.09 | YES |
| D_0__Bacteria;D_1__Firmicutes;D_2__Clostridia;D_3__Clostridiales;D_4__Ruminococcaceae;D_5__Ruminococcaceae UCG-010 | 0.83 | 0.23 | 3.11 | 0.75 | YES | 0.14 | 0.05 | 1.91 | 1.10 | YES |
| D_0__Bacteria;D_1__Firmicutes;D_2__Clostridia;D_3__Clostridiales;D_4__Ruminococcaceae;D_5__Ruminococcus 1 | 0.83 | 0.08 | 2.61 | 1.09 | YES | 0.45 | 0.18 | 2.44 | 1.45 | YES |
| D_0__Bacteria;D_1__Firmicutes;D_2__Clostridia;D_3__Clostridiales;D_4__Lachnospiraceae;D_5__Dorea | 0.78 | 0.07 | 0.72 | 0.09 |  | 0.77 | 0.07 | 0.57 | 0.16 |  |
| D_0__Bacteria;D_1__Firmicutes;D_2__Clostridia;D_3__Clostridiales;D_4__Lachnospiraceae;D_5__Coprococcus 2 | 0.69 | 0.12 | 0.50 | 0.08 |  | 0.21 | 0.10 | 0.12 | 0.04 |  |
| D_0__Bacteria;D_1__Firmicutes;D_2__Erysipelotrichia;D_3__Erysipelotrichales;D_4__Erysipelotrichaceae;D_5__Asteroleplasma | 0.67 | 0.35 | 1.42 | 1.13 |  | 0.06 | 0.30 | 0.10 | 0.01 |  |
| D_0__Bacteria;D_1__Firmicutes;D_2__Clostridia;D_3__Clostridiales;D_4__Clostridiales vadinBB60 group;D_5__uncultured bacterium | 0.65 | 0.22 | 1.35 | 0.40 | YES | 0.15 | 0.25 | 0.20 | 0.08 |  |
| D_0__Bacteria;D_1__Firmicutes;D_2__Clostridia;D_3__Clostridiales;D_4__Ruminococcaceae;D_5__CAG-352 | 0.64 | 0.00 | 0.16 | 0.10 | YES | 0.02 | 0.06 | 0.00 | 0.00 |  |
| D_0__Bacteria;D_1__Proteobacteria;D_2__Gammaproteobacteria;D_3__Aeromonadales;D_4__Succinivibrionaceae;D_5__Ruminobacter | 0.60 | 1.93 | 0.59 | 0.49 |  | 0.00 | 0.00 | 0.05 | 0.00 | YES |
| D_0__Bacteria;D_1__Firmicutes;D_2__Bacilli;D_3__Lactobacillales;D_4__Streptococcaceae;D_5__Streptococcus | 0.56 | 0.15 | 0.65 | 0.26 |  | 0.72 | 0.23 | 0.34 | 0.16 |  |
| D_0__Bacteria;D_1__Spirochaetes;D_2__Spirochaetia;D_3__Spirochaetales;D_4__Spirochaetaceae;D_5__Treponema 2 | 0.54 | 2.64 | 0.30 | 0.41 |  | 0.00 | 0.00 | 0.13 | 0.07 | YES |
| D_0__Bacteria;D_1__Firmicutes;D_2__Clostridia;D_3__Clostridiales;D_4__Ruminococcaceae;D_5__Ruminococcaceae UCG-005 | 0.54 | 0.09 | 1.56 | 0.33 | YES | 0.21 | 0.09 | 0.58 | 0.28 |  |
| D_0__Bacteria;D_1__Bacteroidetes;D_2__Bacteroidia;D_3__Bacteroidales;D_4__Tannerellaceae;D_5__Parabacteroides | 0.51 | 0.13 | 0.56 | 0.35 |  | 1.06 | 0.27 | 0.17 | 0.05 | YES |
| D_0__Bacteria;D_1__Firmicutes;D_2__Clostridia;D_3__Clostridiales;D_4__Lachnospiraceae;D_5__Butyrivibrio | 0.50 | 0.24 | 0.35 | 0.24 |  | 0.03 | 0.03 | 0.12 | 0.07 |  |
| D_0__Bacteria;D_1__Proteobacteria;D_2__Gammaproteobacteria;D_3__Enterobacteriales;D_4__Enterobacteriaceae;D_5__Escherichia-Shigella | 0.47 | 0.13 | 0.58 | 0.29 |  | 0.45 | 0.07 | 0.78 | 0.27 |  |
| D_0__Bacteria;D_1__Cyanobacteria;D_2__Melainabacteria;D_3__Gastranaerophilales;D_4__uncultured bacterium;D_5__uncultured bacterium | 0.45 | 0.11 | 0.45 | 0.21 |  | 0.42 | 0.18 | 0.19 | 0.05 |  |
| D_0__Bacteria;D_1__Elusimicrobia;D_2__Elusimicrobia;D_3__Elusimicrobiales;D_4__Elusimicrobiaceae;D_5__Elusimicrobium | 0.45 | 0.31 | 1.45 | 0.81 |  | 0.07 | 0.36 | 0.74 | 0.55 |  |
| D_0__Bacteria;D_1__Firmicutes;D_2__Bacilli;D_3__Lactobacillales;D_4__Lactobacillaceae;D_5__Lactobacillus | 0.45 | 0.13 | 0.67 | 0.58 |  | 5.37 | 2.04 | 0.43 | 0.28 | YES |
| D_0__Bacteria;D_1__Actinobacteria;D_2__Coriobacteriia;D_3__Coriobacteriales;D_4__Coriobacteriaceae;D_5__Collinsella | 0.43 | 0.07 | 0.41 | 0.06 |  | 1.55 | 0.18 | 1.93 | 1.11 |  |
| D_0__Bacteria;D_1__Bacteroidetes;D_2__Bacteroidia;D_3__Bacteroidales;D_4__Rikenellaceae;D_5__Rikenellaceae RC9 gut group | 0.43 | 0.31 | 0.93 | 0.27 |  | 0.07 | 0.25 | 1.61 | 1.10 | YES |
| D_0__Bacteria;D_1__Firmicutes;D_2__Erysipelotrichia;D_3__Erysipelotrichales;D_4__Erysipelotrichaceae;D_5__Holdemanella | 0.41 | 0.09 | 0.46 | 0.16 |  | 0.13 | 0.06 | 0.36 | 0.20 |  |
| D_0__Bacteria;D_1__Firmicutes;D_2__Erysipelotrichia;D_3__Erysipelotrichales;D_4__Erysipelotrichaceae;D_5__Catenibacterium | 0.40 | 0.05 | 0.29 | 0.08 |  | 0.15 | 0.09 | 0.49 | 0.33 |  |
| D_0__Bacteria;D_1__Firmicutes;D_2__Clostridia;D_3__Clostridiales;D_4__Clostridiaceae 1;D_5__Clostridium sensu stricto 1 | 0.39 | 0.07 | 0.43 | 0.15 |  | 0.64 | 0.58 | 0.20 | 0.06 |  |
| D_0__Bacteria;D_1__Firmicutes;D_2__Clostridia;D_3__Clostridiales;D_4__Ruminococcaceae;D_5__Butyricicoccus | 0.37 | 0.02 | 0.24 | 0.04 | YES | 0.46 | 0.09 | 0.52 | 0.18 |  |
| D_0__Bacteria;D_1__Proteobacteria;D_2__Alphaproteobacteria;D_3__Rhodospirillales;D_4__uncultured;__ | 0.35 | 0.30 | 1.08 | 1.41 |  | 0.06 | 0.00 | 0.06 | 0.04 |  |
| D_0__Bacteria;D_1__Proteobacteria;D_2__Gammaproteobacteria;D_3__Aeromonadales;D_4__Succinivibrionaceae;D_5__uncultured | 0.34 | 0.15 | 0.44 | 0.24 |  | 0.13 | 0.15 | 0.07 | 0.05 |  |
| D_0__Bacteria;D_1__Firmicutes;D_2__Clostridia;D_3__Clostridiales;D_4__Lachnospiraceae;D_5__[Ruminococcus] torques group | 0.34 | 0.06 | 0.37 | 0.08 |  | 0.35 | 0.07 | 0.49 | 0.19 |  |
| D_0__Bacteria;D_1__Firmicutes;D_2__Clostridia;D_3__Clostridiales;D_4__Lachnospiraceae;D_5__[Eubacterium] ruminantium group | 0.32 | 0.09 | 0.22 | 0.04 |  | 0.10 | 0.15 | 0.10 | 0.02 |  |
| D_0__Bacteria;D_1__Firmicutes;D_2__Clostridia;D_3__Clostridiales;D_4__Lachnospiraceae;D_5__Anaerostipes | 0.30 | 0.07 | 0.29 | 0.07 |  | 0.30 | 0.08 | 0.19 | 0.05 |  |
| D_0__Bacteria;D_1__Proteobacteria;D_2__Alphaproteobacteria;D_3__Rhodospirillales;D_4__uncultured;D_5__Azospirillum sp. 47_25 | 0.29 | 0.00 | 0.00 | 0.00 | YES | 0.03 | 0.12 | 0.00 | 0.00 |  |
| D_0__Bacteria;D_1__Firmicutes;D_2__Clostridia;D_3__Clostridiales;D_4__Christensenellaceae;D_5__Christensenellaceae R-7 group | 0.29 | 0.06 | 0.81 | 0.16 | YES | 0.12 | 0.05 | 0.46 | 0.29 | YES |
| D_0__Bacteria;D_1__Bacteroidetes;D_2__Bacteroidia;D_3__Bacteroidales;D_4__Rikenellaceae;D_5__Alistipes | 0.29 | 0.13 | 0.25 | 0.13 |  | 0.24 | 0.10 | 0.15 | 0.14 |  |
| D_0__Bacteria;D_1__Firmicutes;D_2__Negativicutes;D_3__Selenomonadales;D_4__Acidaminococcaceae;D_5__Phascolarctobacterium | 0.28 | 0.22 | 0.22 | 0.13 |  | 0.13 | 0.13 | 0.14 | 0.08 |  |
| D_0__Bacteria;D_1__Firmicutes;D_2__Clostridia;D_3__Clostridiales;D_4__Ruminococcaceae;D_5__Ruminococcaceae UCG-003 | 0.28 | 0.03 | 0.25 | 0.02 |  | 0.15 | 0.02 | 0.24 | 0.04 | YES |
| D_0__Bacteria;D_1__Bacteroidetes;D_2__Bacteroidia;D_3__Bacteroidales;D_4__Muribaculaceae;D_5__metagenome | 0.27 | 0.08 | 1.07 | 0.53 | YES | 0.41 | 0.38 | 2.57 | 3.20 |  |
| D_0__Bacteria;D_1__Bacteroidetes;D_2__Bacteroidia;D_3__Bacteroidales;D_4__Prevotellaceae;D_5__uncultured | 0.26 | 0.26 | 0.93 | 0.86 |  | 0.24 | 0.35 | 0.00 | 0.00 |  |
| D_0__Bacteria;D_1__Firmicutes;D_2__Clostridia;D_3__Clostridiales;D_4__Peptostreptococcaceae;D_5__Romboutsia | 0.26 | 0.13 | 0.06 | 0.02 | YES | 0.01 | 0.00 | 0.04 | 0.02 |  |
| D_0__Bacteria;D_1__Firmicutes;D_2__Clostridia;D_3__Clostridiales;D_4__Ruminococcaceae;D_5__uncultured | 0.25 | 0.07 | 0.67 | 0.12 | YES | 0.06 | 0.02 | 0.42 | 0.28 | YES |
| D_0__Bacteria;D_1__Firmicutes;D_2__Clostridia;D_3__Clostridiales;D_4__Lachnospiraceae;D_5__Lachnospiraceae UCG-004 | 0.25 | 0.03 | 0.13 | 0.02 | YES | 0.42 | 0.04 | 0.27 | 0.12 |  |
| D_0__Bacteria;D_1__Firmicutes;D_2__Clostridia;D_3__Clostridiales;D_4__Ruminococcaceae;__ | 0.24 | 0.05 | 0.56 | 0.07 | YES | 0.12 | 0.04 | 0.22 | 0.08 |  |
| D_0__Bacteria;D_1__Firmicutes;D_2__Clostridia;D_3__Clostridiales;D_4__Ruminococcaceae;D_5__Ruminococcaceae UCG-013 | 0.24 | 0.10 | 0.29 | 0.04 |  | 0.15 | 0.08 | 0.55 | 0.39 |  |
| D_0__Bacteria;D_1__Firmicutes;D_2__Clostridia;D_3__Clostridiales;D_4__Clostridiales vadinBB60 group;D_5__metagenome | 0.23 | 0.39 | 0.33 | 0.22 |  | 0.01 | 0.02 | 0.20 | 0.00 | YES |
| D_0__Bacteria;D_1__Proteobacteria;D_2__Gammaproteobacteria;D_3__Enterobacteriales;D_4__Enterobacteriaceae;D_5__Klebsiella | 0.22 | 0.25 | 0.05 | 0.08 |  | 0.01 | 0.01 | 0.76 | 0.00 | YES |
| D_0__Bacteria;D_1__Firmicutes;D_2__Clostridia;D_3__Clostridiales;D_4__Ruminococcaceae;D_5__Ruminococcaceae NK4A214 group | 0.19 | 0.03 | 0.33 | 0.05 | YES | 0.08 | 0.06 | 0.18 | 0.12 |  |
| D_0__Bacteria;D_1__Proteobacteria;D_2__Gammaproteobacteria;D_3__Pasteurellales;D_4__Pasteurellaceae;D_5__Haemophilus | 0.19 | 0.06 | 0.03 | 0.01 | YES | 0.58 | 0.24 | 0.19 | 0.25 |  |
| D_0__Bacteria;D_1__Firmicutes;D_2__Clostridia;D_3__Clostridiales;D_4__Lachnospiraceae;D_5__Coprococcus 3 | 0.18 | 0.02 | 0.14 | 0.01 | YES | 0.06 | 0.03 | 0.13 | 0.04 | YES |
| D_0__Bacteria;D_1__Firmicutes;D_2__Clostridia;D_3__Clostridiales;D_4__Lachnospiraceae;D_5__[Eubacterium] ventriosum group | 0.17 | 0.09 | 0.18 | 0.10 |  | 0.04 | 0.06 | 0.03 | 0.01 |  |
| D_0__Bacteria;D_1__Firmicutes;D_2__Clostridia;D_3__Clostridiales;D_4__Lachnospiraceae;D_5__Coprococcus 1 | 0.17 | 0.02 | 0.19 | 0.03 |  | 0.08 | 0.03 | 0.11 | 0.02 |  |
| D_0__Bacteria;D_1__Firmicutes;D_2__Erysipelotrichia;D_3__Erysipelotrichales;D_4__Erysipelotrichaceae;D_5__Erysipelotrichaceae UCG-003 | 0.17 | 0.04 | 0.07 | 0.04 | YES | 0.18 | 0.06 | 0.04 | 0.02 | YES |
| D_0__Bacteria;D_1__Cyanobacteria;D_2__Melainabacteria;D_3__Gastranaerophilales;__;__ | 0,16 | 0,21 | 0,11 | 0,07 |  | 0,04 | 0,18 | 0,29 | 0,59 |  |
| D_0__Bacteria;D_1__Firmicutes;D_2__Clostridia;D_3__Clostridiales;D_4__Clostridiales vadinBB60 group;D_5__gut metagenome | 0,16 | 0,05 | 0,41 | 0,10 |  | 0,02 | 0,04 | 0,06 | 0,02 |  |
| D_0__Bacteria;D_1__Firmicutes;D_2__Clostridia;D_3__Clostridiales;D_4__Ruminococcaceae;D_5__Ruminiclostridium 6 | 0,15 | 0,02 | 0,20 | 0,06 |  | 0,05 | 0,05 | 0,14 | 0,06 |  |
| D_0__Bacteria;D_1__Proteobacteria;D_2__Deltaproteobacteria;D_3__Desulfovibrionales;D_4__Desulfovibrionaceae;D_5__Desulfovibrio | 0,14 | 0,05 | 0,23 | 0,31 |  | 0,09 | 0,05 | 0,03 | 0,02 |  |
| D_0__Bacteria;D_1__Actinobacteria;D_2__Coriobacteriia;D_3__Coriobacteriales;D_4__Eggerthellaceae;D_5__Senegalimassilia | 0,14 | 0,02 | 0,15 | 0,03 |  | 0,20 | 0,03 | 0,24 | 0,07 |  |
| D_0__Bacteria;D_1__Firmicutes;D_2__Clostridia;D_3__Clostridiales;D_4__Ruminococcaceae;D_5__Ruminiclostridium 9 | 0,13 | 0,01 | 0,14 | 0,02 |  | 0,07 | 0,01 | 0,24 | 0,16 | YES |
| D_0__Bacteria;D_1__Epsilonbacteraeota;D_2__Campylobacteria;D_3__Campylobacterales;D_4__Campylobacteraceae;D_5__Campylobacter | 0.09 | 0.17 | 0.62 | 0.24 | YES | 0.74 | 0.13 | 0.43 | 0.17 | YES |
| D_0__Bacteria;D_1__Verrucomicrobia;D_2__Verrucomicrobiae;D_3__Verrucomicrobiales;D_4__Akkermansiaceae;D_5__Akkermansia | 0.08 | 0.04 | 0.16 | 0.07 |  | 0.20 | 0.14 | 0.00 | 0.00 | YES |
| D_0__Bacteria;D_1__Bacteroidetes;D_2__Bacteroidia;D_3__Bacteroidales;D_4__Prevotellaceae;D_5__Prevotella 7 | 0.02 | 0.02 | 0.03 | 0.02 |  | 0.03 | 0.03 | 0.05 | 0.00 |  |
| D_0__Bacteria;D_1__Firmicutes;D_2__Bacilli;D_3__Lactobacillales;D_4__Enterococcaceae;D_5__Enterococcus | 0.01 | 0.02 | 0.03 | 0.00 |  | 0.02 | 0.06 | 0.17 | 0.04 | YES |
